# Ga Doping Enables Precision Alloy‐to‐Wire Regulation: Synergistic Enhancement of the Mechanical Properties of CuSn Alloy and the Superconducting Properties of Nb_3_Sn

**DOI:** 10.1002/advs.202515530

**Published:** 2026-02-25

**Authors:** Dazhuo Song, Juntao Zou, Jiayue Zhang, Xinhang Liang, Mengyu Shan, Yifan Liu, Jingqi Feng, Tong Dang, Lin Shi, Yuxuan Wang, Yuchen Song, Rong Fei, Shaodong Sun, Zhe Zhang, Lei Zhu, Lixing Sun

**Affiliations:** ^1^ Engineering Research Center of Conducting Materials and Composite Technology Ministry of Education, Shaanxi Key Laboratory of Electrical Materials and Infiltration Technology Shaanxi Laboratory of Advanced Materials School of Materials Science and Engineering Xi'an University of Technology Xi'an P. R. China; ^2^ Faculty of Humanities and Foreign Languages Xi'an University of Technology Xi'an P. R. China; ^3^ Faculty of Computer Science and Engineering Xi'an University of Technology Xi'an P. R. China

**Keywords:** copper alloys, element doping, slip and twinning, strength–ductility, superconducting properties

## Abstract

Cu–Sn alloy is a key raw material used in the preparation of Nb_3_Sn superconducting wires using the bronze method. The mechanical properties of this Cu–Sn alloy are directly responsible for determining the properties of the superconducting wires. The superconducting properties of Nb_3_Sn can be significantly improved by Ga doping. However, the effect of Ga doping and the amount thereof on the properties of Cu–Sn alloys has rarely been reported. In this study, the distribution of Ga and its impact on the mechanical properties of Cu–Sn alloys are investigated via experiments and simulations. The results indicate that Cu and Ga readily produce strong electron exchange and a stable solid‐solution structure, leading to Ga superiority in the solid‐solution competition with Sn and to δ phase segregation. For the first time, a 1.0 wt.% Ga‐added Cu–Sn alloy exhibiting an excellent elongation of 100.8% was successfully prepared. Further investigation revealed that with increasing Ga content, the activation of planar fault slip systems becomes more difficult, the volume fraction of planar fault structures gradually decreases, and the dislocation dissociation distance decreases as cross‐slip occurs. The simulation results revealed that as Ga doping increases, the matrix stacking fault energy increases and the deformation mechanism shifts from being dominated by twinning to a balanced combination of slip and twinning, which is the primary mechanism for the synergistic enhancement of strength and ductility in the alloy. In addition, Ga doping significantly elevated the superconducting transition temperature of Nb_3_Sn by approximately 0.85 K and improved the critical current density. This study innovatively demonstrates that compared to the front‐end Cu–Sn alloy, the addition of 1.0 wt.% Ga achieves synergistic property enhancement of the back‐end Nb_3_Sn superconducting wire. This study provides both a theoretical and experimental foundation for the preparation of Cu–Sn alloys with elevated properties for use in Nb_3_Sn superconducting wires.

## Graphical abstract

By adjusting Ga's doping in Cu–Sn alloy, an ultra‐high elongation of 100.8% is achieved in first time. Ga forms a stable solid solution structure and a stronger electron exchange with Cu. This enhances strength‐ductility synergistically by tuning SFE to balance twinning and slip. Ga doping also increased the *T*_c_ and *J*_c_ of Nb_3_Sn, which suggests a new strengthening strategy for superconductors.

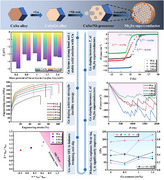

## 1 Introduction

Nb_3_Sn superconducting wires—as the primary conductor material for high‐current cable‐in‐conduit configurations in the international thermonuclear experimental reactor (ITER) project—face critical reliability challenges rooted in their Cu–Sn matrix properties [1, 2]. The manufacturing process itself presents inherent vulnerabilities in that the multistage bundle drawing required for kilometer‐scale wire production generates localized stress concentrations that induce structural failure [3]. Furthermore, during operation, these conductors must withstand intense electromagnetic forces generated by current loads of more than 68 kA, creating additional risks of Nb_3_Sn filament fracture [4]. These compound mechanical demands arising from both fabrication stresses (tensile‐dominated) and operational loads (bending/torsional) necessitate the dual requirement of simultaneous enhancement of strength and ductility for the Cu–Sn alloy. Addressing this strength–ductility paradox through advanced Cu–Sn alloy design has emerged as the fundamental solution to prevent fracture propagation across both production and application phases [5, 6].

In Cu–Sn alloys used as raw materials for Nb_3_Sn superconducting wires, Sn forms a supersaturated solid solution in the Cu matrix to maximize its role as a sufficient Sn source during Nb_3_Sn phase formation. This places high demands on the method for solidification of the Cu–Sn alloy in that segregation must be suppressed and dispersed as far as possible while increasing the solid solution content of Sn. The traditional casting method introduces a large amount of macrosegregation, which promotes brittleness in the alloy. Furthermore, uncoordinated deformation between grains is not conducive to large plastic deformation of the alloy [7]. With the development of selective laser melting (SLM) technology, studies have demonstrated that the extremely fast solidification rate of SLM can yield fine micron‐sized grains, thus resolving the macrosegregation problem. The significant improvement in ultimate tensile strength (UTS) of more than 600 MPa is easily elucidated by the Hall‐Petch relationship. However, this improvement in UTS is at the expense of ductility (ε ≤ 30%), which is obviously not in line with the development concept of high‐ductility Cu–Sn alloys [8]. Osaka Alloy of Japan first proposed the “Mizuta” method for preparing Cu–Sn alloys with uniform Sn distribution. Compared with traditional casting technology, the “Mizuta” method converts the three‐dimensional spatial distribution of elements into a one‐dimensional‐plane problem, thereby improving the uniformity of the structure and thus improving the ductility of the alloy [9]. In a recent report, a Cu–Sn alloy was strengthened by adding more than 3.0 wt.% Zn. The Zn was confirmed to play a role in solid‐solution strengthening in the Cu matrix, thereby improving the ability of the alloy to resist the Lorentz force under high magnetic field strength. This method also furnishes ideas for the subsequent production of Cu–Sn alloys for Nb_3_Sn superconducting wires [4].

In recent years, research on improving the ductility of Cu–Sn alloys has focused on regulating the strengthening mechanism [10]. In our previous work, the addition of 0.3 wt.% Ti increased the strength of the Cu–15Sn alloy by 100 MPa; however, elongation was not significantly improved. This was attributed to the fact that grain refinement shortened the dislocation slip distance, thereby accelerating the decomposition of all dislocations and leading to the nucleation and expansion of twin boundaries (TBs) [11]. In fact, ductility improvement depends to a certain extent on the long‐range slip of dislocations, which produces an essentially inverted relationship between the strength and ductility of the alloy [12]. From the perspective of solidification, balancing the dominance of slip and twinning is difficult. Storing dislocations by suppressing dynamic recovery is a common processing method for face‐centered cubic (FCC) metals. Lu et al. [13] introduced high‐density TBs into Cu through plastic processing. In the subsequent plastic deformation, the nanoscale TBs both hindered dislocation movement and allowed dislocation storage to promote work hardening, achieving a synergistic enhancement in strength–ductility. In response to the poor ductility of magnesium alloys, researchers have found that {102} TBs can eliminate basal dislocations and promote the reaction between TBs and dislocations to form slippable 60° dislocations through large plastic deformation, thereby significantly improving the ductility of magnesium alloys [14]. Note that the interaction between dislocations and TBs can improve ductility. However, because slip is conducive to ductility, when competition with typical twinning behavior occurs, the matrix must be in a state where dislocations are difficult to cross‐slip and are about to fail. In other words, the claimed improvement in ductility can reasonably be inferred to have not actually reached the plastic limit. Therefore, mechanical properties are often reliant on almost harsh processing methods [15].

The slip–twinning tradeoff in alloy strengthening has thus drawn considerable research attention to its fundamental mechanisms. When a full dislocation decomposes into two Shockley partial dislocations in an FCC crystal, the energy barrier that needs to be overcome is called stacking fault energy (SFE). The magnitude of SFE determines the width of the dislocation dissociation, and the two are inversely proportional [16]. Notably, the larger the distance between two partial dislocations, the more difficult it is to form a full dislocation again. This leads to a significant increase in the critical stress for cross‐slip because the dislocation is more inclined toward plane‐slip than cross‐slip. More importantly, SFE is generally believed to be directly proportional to the critical shear stress of the twinning of the alloy. In short, as an intrinsic parameter of the material, SFE directly affects the slip mode and work‐hardening ability [17]. SFE can be precisely controlled by chemical doping. The alloy usually exhibits a higher SFE, which leads to a significant increase in the tendency for dislocation cross‐slip, accelerated dislocation annihilation, and decreased work‐hardening ability [18]. The FeMnCoCr alloy reduces SFE by introducing interstitial N atoms, thereby increasing the yield strength to 1.8 GPa and elongation to 11.6% at low temperatures [19]. Similarly, pure copper exhibits a plastic limit and shear band formation tendency owing to its higher SFE, whereas the UTS and elongation of Cu–Al [20] and Cu–Zn [21] alloys are synergistically enhanced. The same mechanism can also explain the improvement in ductility of the Cu–Sn alloys. In another case, the SFE of nickel‐based superalloys was generally high and further increased with increasing temperature. Here, the creep mechanism was dominated by superdislocation shear, with a lack of stacking fault (SF) strengthening, resulting in a decrease in creep resistance [22]. In summary, the rational control of SFE requires a balance between the slip mechanism and twinning behavior, and composition optimization brought about by element doping is the most reasonable and reliably effective approach.

For Cu–Sn alloys, the type of doping element directly affects the properties of Nb_3_Sn superconducting wires. The bronze method is the mainstream process for producing Nb_3_Sn superconducting wires. Research on improving the superconducting properties of Nb_3_Sn by doping with Ti and Ta has furnished ideas for overcoming the property limits of Nb_3_Sn [23, 24]. Similar elements, including Al, Mg, Zn, Zr, Ag, Au, Hf, Ge, and In, have been added to the Cu–Sn matrix. These elements improve the superconducting properties of Nb_3_Sn superconductors by regulating the grain size and growth rate of Nb_3_Sn. As a notable exception, Ga has been found to directly enter the Nb_3_Sn layer and replace Nb and Sn atoms, thereby improving the superconducting properties [23, 24, 25, 26, 27, 28, 29]. Sekine et al. added 4.0 wt.% Ga to a Cu–5Sn alloy, thereby increasing the superconducting transition temperature (*T_c_*) by 0.3–0.5 K and upper critical magnetic field strength (*H_c2_*) by 2–4 T [23, 28]. On this basis, Rudziak et al. increased the Sn addition to 13.0–14.0 wt.% and presented a detailed discussion on the Ga additions of 4.0 and 8.0 wt.%. The results showed that Ga addition improved the critical current density (*J_c_*) of Nb_3_Sn, but owing to the core fracture, no obvious regularity between the Ga content and superconducting properties was observed. For Cu–14Sn alloys close to a saturated solid solution, such a high Ga addition is highly likely to cause solute segregation, resulting in brittle fracture of the Cu–Sn matrix [29]. Although the strengthening mechanism of Ga on Nb_3_Sn is clear, few reports exist on the effect of Ga doping on the properties of Cu–Sn alloys. Notably, the results of relevant theoretical calculations have shown that low‐content Ga doping can increase the SFE of Cu alloys, which may lead to changes in the deformation mechanism of the originally low‐SFE Cu–Sn alloy. Therefore, regulation of the Ga content to control the mechanical properties of the alloy is expected to overcome the property limits of the Cu–Sn alloy as a superconducting wire substrate [10, 30]. In the face of the increasingly high property requirements of Cu–Sn alloys in the production process and service environment of Nb_3_Sn superconducting wires, a systematic study of the effects of different Ga doping amounts on the microstructure and properties of Cu–Sn alloys and further verification of the effect of Ga on the superconducting properties of Nb_3_Sn at the optimal addition amount is an issue that needs to be urgently addressed and is the focus of this study.

In this study, the microstructural evolution of Cu–Sn alloys doped with different Ga contents was investigated, and the competitive solid‐solution behavior between Sn and Ga was elucidated through first‐principles calculations. The mechanical properties of Cu–Sn alloys with different Ga additions were further examined, and the deformation structures of the alloys were characterized in detail. The Ga‐mediated strengthening mechanism was also revealed via computational approaches. Finally, Ga‐doped Nb_3_Sn superconductors were fabricated, and their superconducting properties were explored through a combination of experimental and theoretical methods. This study demonstrates the significant potential of Ga doping in simultaneously improving the superconducting properties of Nb_3_Sn and enhancing the strength and ductility of Cu–Sn alloys. The findings provide a theoretical foundation for a deeper understanding of the strengthening mechanisms and processing strategies for doped Cu–Sn alloys; an experimental basis for their application; and a viable pathway toward the efficient, reliable, and safe use of Nb_3_Sn superconducting wires in controlled nuclear fusion engineering.

## 2 Results and Discussion

### 2.1 Microstructures of the Alloy Before and After Solution Treatment

**FIGURE 1:**
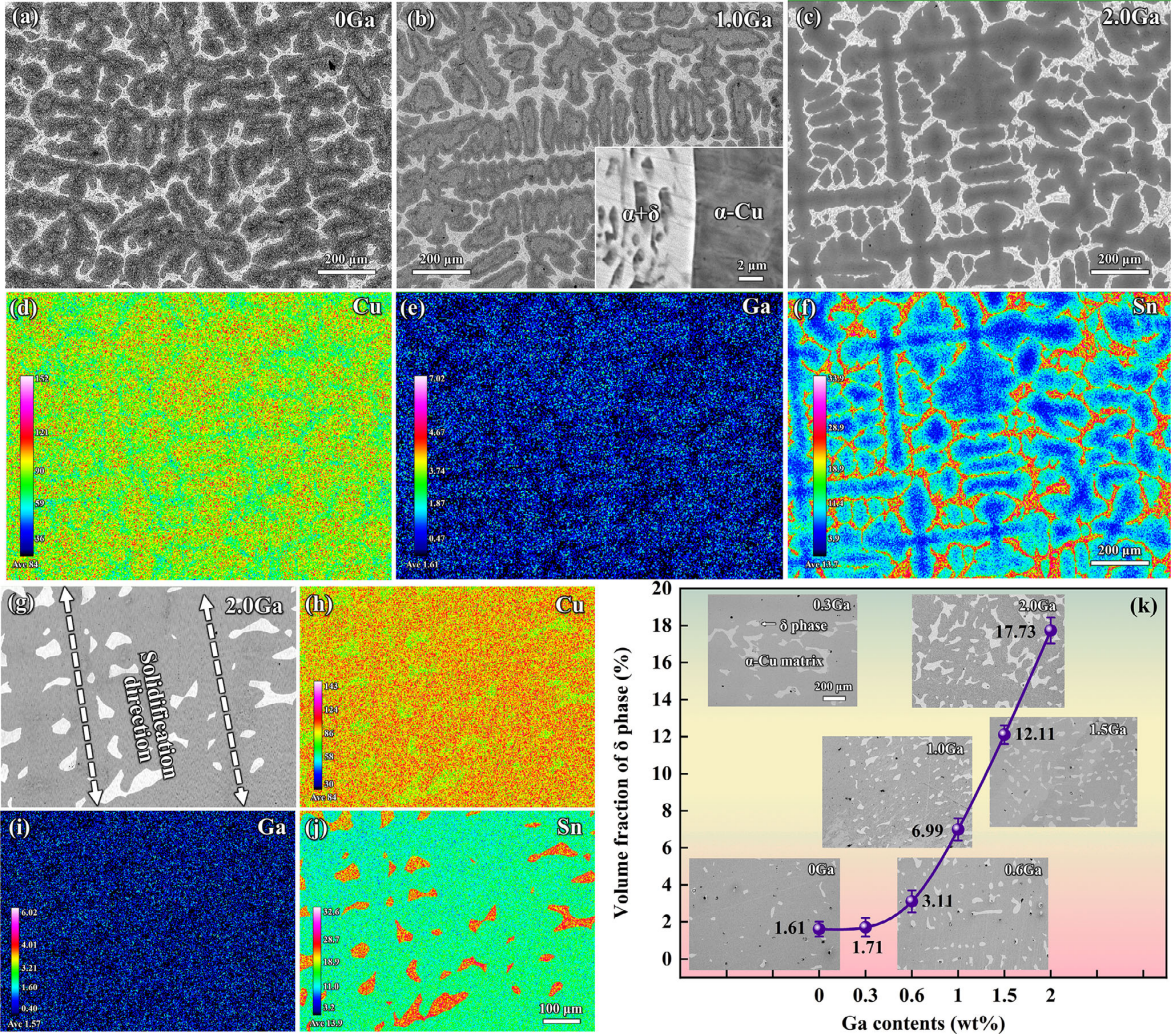
Microstructure of the Cu–Sn alloy with different Ga contents. (a–c) As‐cast structure, (d–f) EPMA results for the as‐cast Cu–15Sn–2Ga alloy, (g–j) EPMA results for the Cu–15Sn–2Ga alloy after solution treatment, and (k) relationship between Ga content and δ phase volume fraction after solution treatment.

Figure 1a–c present the microstructures of alloys with different Ga contents after directional solidification; the micrographs show that the as‐cast alloys all have typical dendritic structures. Using ICP‐OES, the chemical composition of the as‐cast Cu–15Sn–0.3Ga alloy was determined to be 14.677 wt.% Sn and 0.295 wt.% Ga, with Cu making up the balance. The deviation between the actual and nominal composition is attributed to Sn burnout during the smelting process. Compared with Sn, Ga burnout is significantly lower. The electron probe X‐ray microanalyzer (EPMA) analysis of the Cu–15Sn–2Ga alloy (Figure 1d–f) shows that the proportion of Sn in the matrix is significantly lower than that between the dendrites, and Ga is mainly distributed in the dendrite trunk, indicating that Ga is more easily dissolved in Cu than Sn. The latest Cu–Sn [31] and Cu–Ga [32] phase diagrams clearly show that the room‐temperature solubility of Ga in Cu is greater than that of Sn in Cu. According to the ratio of elements, the α + δ eutectoid structure between the dendrites is the typical phase structure of the Cu–Sn alloy. A change in Ga content does not induce a significant change in dendrite parameters and phase composition [7, 10, 11].

Solution treatment of alloys prone to segregation is an important prerequisite for discussing their properties. Figure 1g–j show the microstructure and elemental distribution of the Cu–15Sn–2Ga alloy after solution treatment at 650°C for 72 h. The α+δ eutectoid structure can be observed to have been completely eliminated, and undissolved cellular δ phase is distributed in the α‐Cu supersaturated solid solution, which corresponds to the projection of rod‐shaped or granular δ phases onto the plane in three‐dimensional space. The distribution of the δ phase after solution treatment has a certain regularity. This is because directional solidification promotes a dendrite array with preferential orientation growth. Solution treatment leads to the balancing of element concentrations in each region of the alloy; however, the δ phase is not completely dissolved between the dendrites and still retains the orderly distribution of directional solidification structural characteristics [33]. The statistical results for the volume fraction of the δ phase show that with increasing Ga content, the volume fraction of the δ phase gradually increases, and the growth rate is positively proportional to the Ga content.

### 2.2 Interaction Between Ga and Sn

#### 2.2.1 Effect of Solution Treatment on Elemental Distribution

**FIGURE 2:**
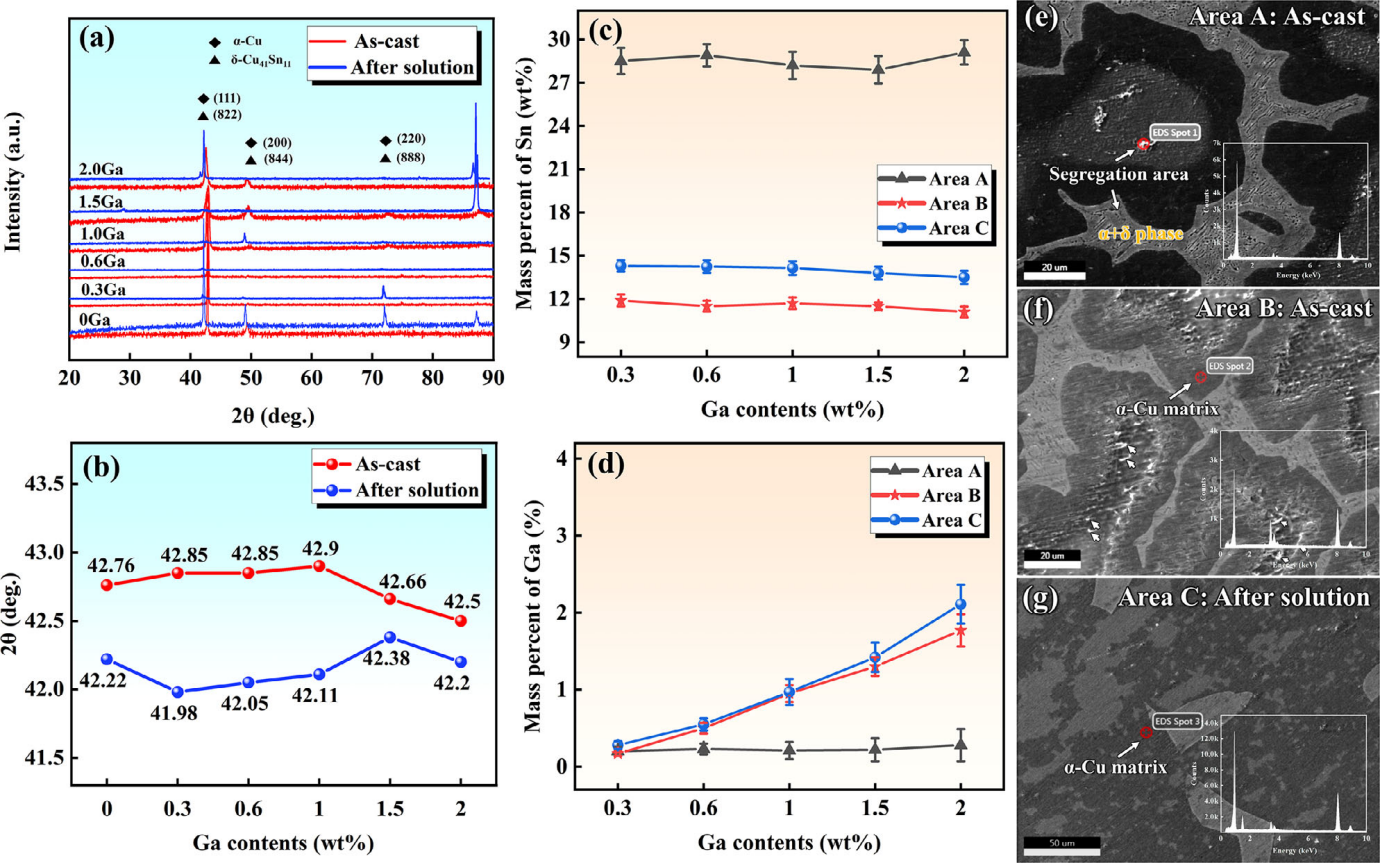
(a) XRD patterns of different alloys in the 2θ range of 20–90°; (b) change in 2θ of the (111) peak with Ga content; (c) and (d) collection results of the mass percent of Sn and Ga in areas A, B, and C for different alloys under EPMA mode; and (e–g) collection positions of areas A, B, and C.

Figure 2a,b show the X‐ray diffraction (XRD) results for the alloys with different Ga contents. Before and after solid‐solution treatment, only elemental diffusion occurs in the alloy, with no phase change occurring. One can reasonably infer that Ga exists mainly in the form of a solid solution in the matrix. Figure 2b shows the statistical results of the 2θ values of the diffraction (111) peaks of alloys with different Ga contents. The 2θ of the as‐cast alloy initially remains essentially stable as the Ga content increases, but when the Ga content exceeds 1.0 wt.%, 2θ decreases slightly, indicating a significant increase in interplanar spacing. After sufficient solution treatment, 2θ decreases significantly overall. However, as the Ga content further increases, 2θ increases slightly, indicating the desolvation of solute atoms. This explains the linear relationship between the degree of segregation and Ga content observed in the statistical results shown in Figure 1k. A similar phenomenon was observed in a study on Mg–Al–Zn–Gd alloys, which was attributed to the repulsion of Ga atoms on Sn atoms [34]. Figure 2c–g show the distributions of Sn and Ga in different collection areas under the EPMA mode. The results show that the chemical composition of the bright white phase in area A is consistent with the elemental ratio of the Cu_41_Sn_11_ intermetallic compound. As the Ga content increases, the Sn content in area A remains unchanged. This relates to the fixed elemental composition of the intermetallic compound. In area B, as the Ga content increases, the Sn content remains essentially unchanged. Note that in the α‐Cu matrix after solid solution in area C, the Sn content slowly decreases with Ga addition. The completely different change patterns of Sn and Ga indicate that Ga competes with Sn for solid solution.

#### 2.2.2 Calculation Based on First Principles

The atomic radii of Cu, Ga, and Sn are 0.128, 0.122 (approximately 4.7% smaller than Cu), and 0.141 nm (10.2% larger than Cu), respectively. According to the Hume‐Rothery rule, the difference in atomic radius between the solute and matrix must be less than 15%. Both Sn and Ga meet the atomic size requirements and can form a substitutional solid solution [35]. A recent study reported that in multi‐principal element‐strengthened solid‐solution alloys, the repulsive relationship between different solute elements leads to differences in solid solubility, and in supersaturated solid solutions, competition between Sn and Ga is also inevitable [36]. First, because the atomic size of Ga is closer to that of Cu and its valence state is lower, the electron concentration is subjected to less disturbance. Second, the difference in the solid solubilities of Sn and Ga in Cu is related to the intrinsic parameters of the solute atoms doped into the Cu unit cell, parameters such as the formation energy of the solute atoms replacing Cu atoms to form a stable solid solution structure [37]. The formation energy *E_f_* represents the energy released or absorbed by different solute atoms when they enter a single crystal cell to form a solid solution and can be expressed as follows [38]:

(1)

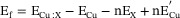

where *X* denotes Sn and Ga, *E_Cu:X_* is the total energy of solute atoms dissolved into the unit cell, *𝐸_Cu_* is the total energy of the pure Cu system with undoped solute atoms the same size as those of the doped system, 𝐸_X_ is the energy of a single solute atom under vacuum, *E'_Cu_* is the energy of a single Cu atom under vacuum, and *n* is the number of doped solute atoms. Because the doping of only one alloy atom is considered (*n* = 1), a negative *E_f_* indicates that the energy released by forming a solid solution can exist stably. The more negative the *E_f_*, the easier a stable structure will form. The formation energy calculated for the same mass ratio of Sn and Ga entering the Cu unit cell to form a solid‐solution structure are presented in Table 1 and Figure 4a. When the mass percent of Ga and Sn is the same, the solid solution of Ga atoms entering the Cu unit cell has a more negative formation energy, indicating that, compared with Sn, Ga is more likely to enter the Cu unit cell to replace Cu atoms to form a stable solid solution.

**TABLE 1:** Formation energy of Cu–(Ga, Sn) solid solutions with different solute mass percentages.

Mass percentage	Supercell	E_Cu:X_ (eV)	E_Cu_/eV	E_X_ (eV)	E'_Cu_ (eV)	E_f_ (eV)
0.4 wt.%	Cu_383_Sn_1_	−565614.52	−566995.32	−91.71	−1476.56	−4.05
	Cu_255_Ga_1_	−378574.46	−377996.04	−2050.02		−4.96
0.8 wt.%	Cu_215_Sn_1_	−317554.18	−318936.00	−91.71		−3.03
	Cu_143_Ga_1_	−213199.92	−212622.60	−2050.02		−3.86
1.0 wt.%	Cu_191_Sn_1_	−282113.39	−283495.26	−91.71		−2.88
	Cu_107_Ga_1_	−160043.54	−159466.11	−2050.02		−3.97
1.7 wt.%	Cu_107_Sn_1_	−158085.86	−159466.12	−91.71		−3.73
	Cu_63_Ga_1_	−95076.46	−94498.42	−2050.02		−4.58
3.4 wt.%	Cu_47_Sn_1_	−69492.86	−70874.38	−91.71		−3.33
	Cu_31_Ga_1_	−47826.32	−47249.42	−2050.02		−3.44

**FIGURE 3:**
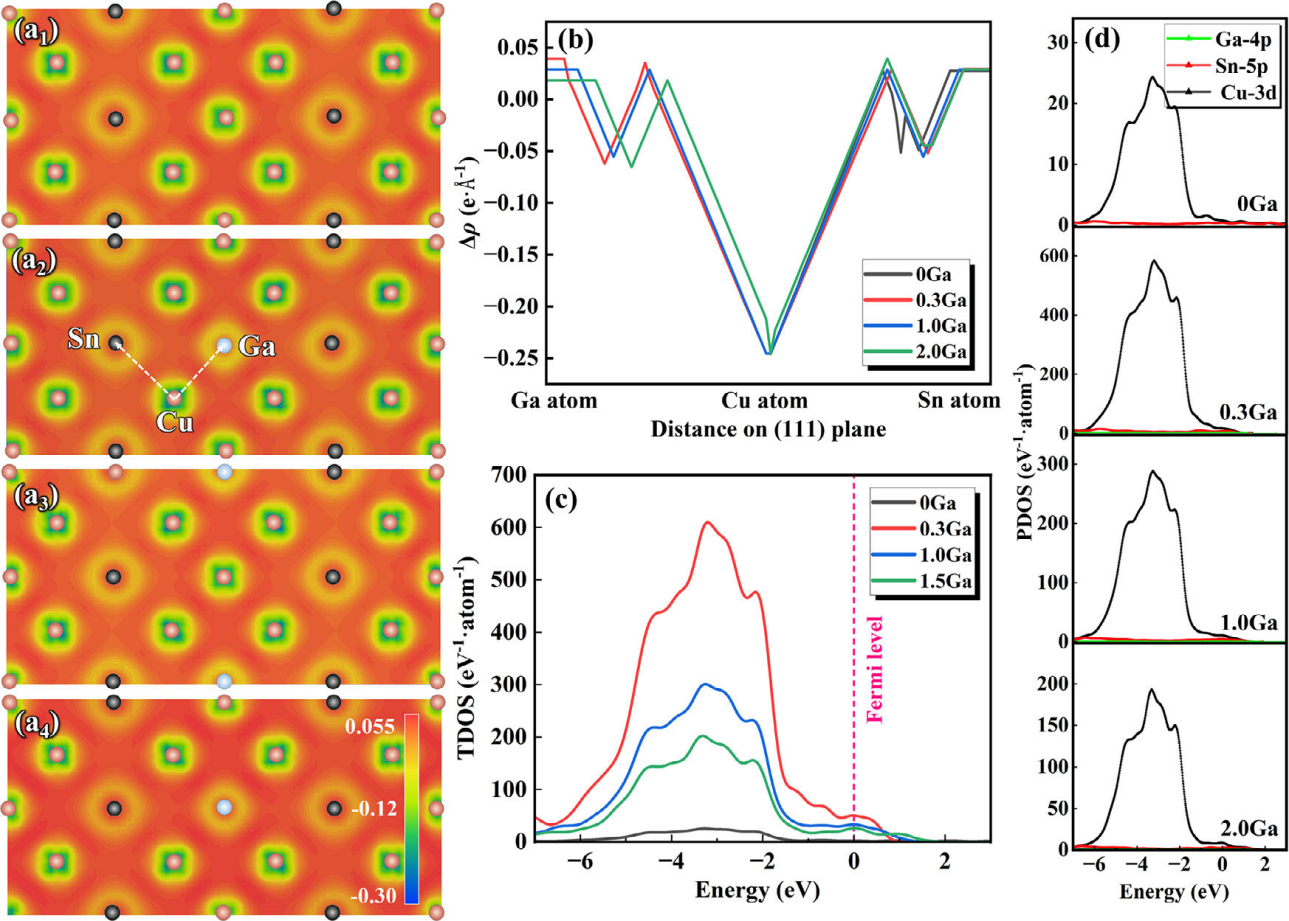
(a) Differential charge–density maps for different Ga contents alloys, (b) charge transfer between atoms, (c) total density of states of the pure and doped systems, and (d) partial densities of states.

The redistribution of charge after the addition of Ga into the Cu–Sn alloy can be reflected by calculating the differential charge–density (Δ*ρ* = *ρ*_Cu‐Sn‐Ga_ − *ρ*_Cu‐Sn_), thereby elucidating its solidification characteristics and mechanical properties [39]. As shown in Figure 3a, Δ*ρ* > 0 in the yellow area and Δ*ρ* < 0 in the blue area indicate the accumulation and loss of charge, respectively. The statistical results for the change in Δ*ρ* along the atomic close‐packed direction shown in Figure 3b reveal that Ga and Sn both gain electrons, whereas Cu loses electrons. As the Ga content increases, the Δ*ρ* value between Cu and Ga gradually decreases but remains higher than that between Cu and Sn. This indicates that the charge transfer between Ga and Cu is more intense than that between Sn and Cu. Furthermore, the addition of Ga introduces more free electrons, altering the local charge distribution and causing the charge transfer between Cu and Ga to tend toward equilibrium with increasing Ga content. Figure 3c shows the total state density of the alloy; the valence band of the alloy is mainly concentrated between −8.0 and 2.0 eV. Ga addition significantly changes the state density distribution of the alloy near the Fermi level, thus affecting the localization and delocalization of the electronic state, which has an important impact on the mechanical properties of the alloy. Specifically, when the Ga content increases from 0 to 0.3 wt.%, the density of states (*N_EF_*) near the Fermi level increases from 2.27 to 50.54. This leads to an increase in the regional charge–density, thereby enhancing the interactions between the atoms. The latest research claims that this change is conducive to dislocation movement. This is because the slip of dislocations requires a relative displacement between atoms, and the enhancement in interatomic interactions can provide a driving force for dislocation movement [40]. When the Ga content increases to 1.0 wt.%, *N_EF_* reverts to 30.37, indicating that electron filling tends to be stable. *N_EF_* maintains a high value. Here, the moderate localization of the electronic state not only retains the low‐resistance characteristics of dislocation movement but also delays necking through dynamic recovery, enabling the alloy to evenly coordinate strain during deformation. As demonstrated by the density of states results shown in Figure 3d, no hybridization peak is present, indicating that the electrons have high mobility at the Fermi level, which is consistent with typical metal conduction behavior. Owing to the low concentration of Ga in the alloy, the orbital state density of Ga is extremely low. The 4p orbital of Ga is located in other energy regions, close to the Fermi level of −1.0 to 1.0 eV, whereas the peak at −3.0 eV is mainly dominated by the 3d orbital of Cu. The Ga 4p orbital is likely highly localized, lacking effective overlap with the neighboring Cu–3d and Sn–5p orbitals. This results in electron localization around the Ga atom and does not contribute to bonding hybridization. This suggests that the system is dominated by metallic bonds, with Ga atoms not entering key positions in the lattice to form a secondary phase, resulting in a weak contribution from their orbitals toward the total density of states [41].

**FIGURE 4:**
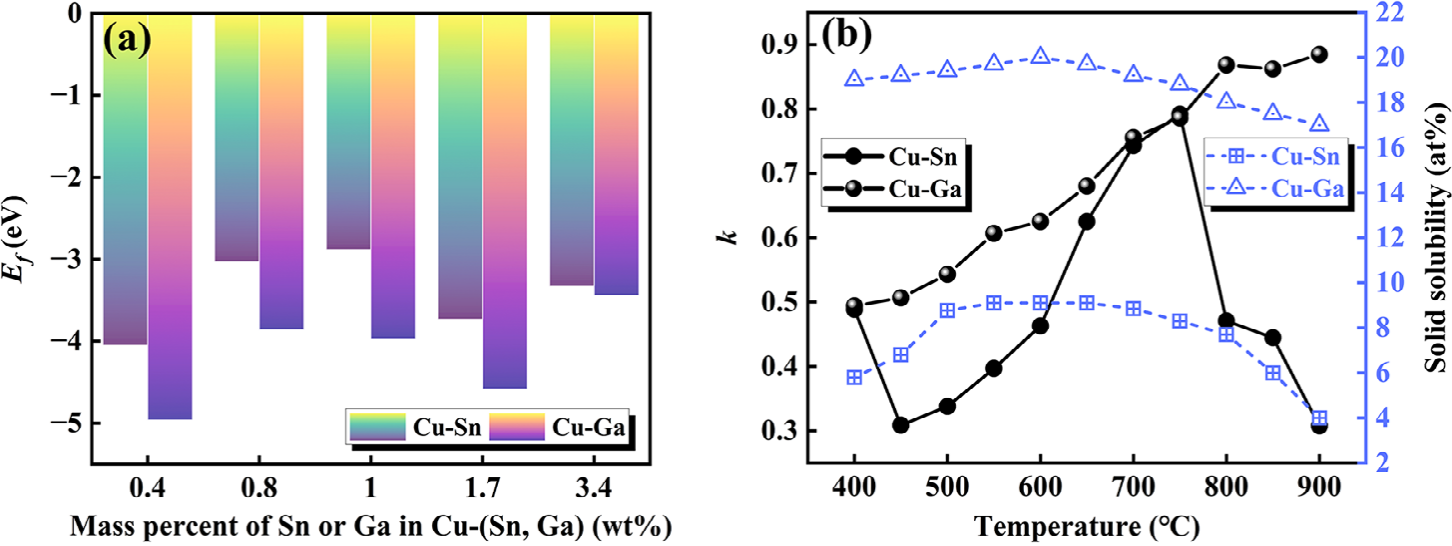
Solute atom solid solubility in Cu–(Sn, Ga) systems. (a) Variation trend for formation energy with different mass percentages, and (b) equilibrium partition coefficient (*k*) and solid solubility of solute atoms at different temperatures.

As shown in Figure 4b, the equilibrium partition coefficient (*k*) and solid solubility of solute atoms at different temperatures were calculated according to recent reports on Cu–Sn [31] and Cu–Ga [32] binary phase diagrams. The results show that both solutes in the alloy tend to be enriched in the liquid phase (*k* < 1), exhibiting positive segregation behavior. A previous study reported a solidification temperature for Cu–15Sn alloy of approximately 798°C [10]. At this temperature, *k_Sn_* deviates by more from 1 compared to *k_Ga_*, indicating that the segregation tendency of Sn in the liquid phase is significantly higher than that of Ga. Furthermore, the comparison results for solid solubility also confirm that the solid solubility of Ga in Cu is higher than that of Sn at the same temperature. In summary, Ga has the advantage of competing with Sn for solid solution, resulting in a reduction in the originally limited substitution sites of Sn. As the Ga content increases, Sn—which cannot be solid‐dissolved—segregates at the grain boundaries.

### 2.3 Tensile Properties

**FIGURE 5:**
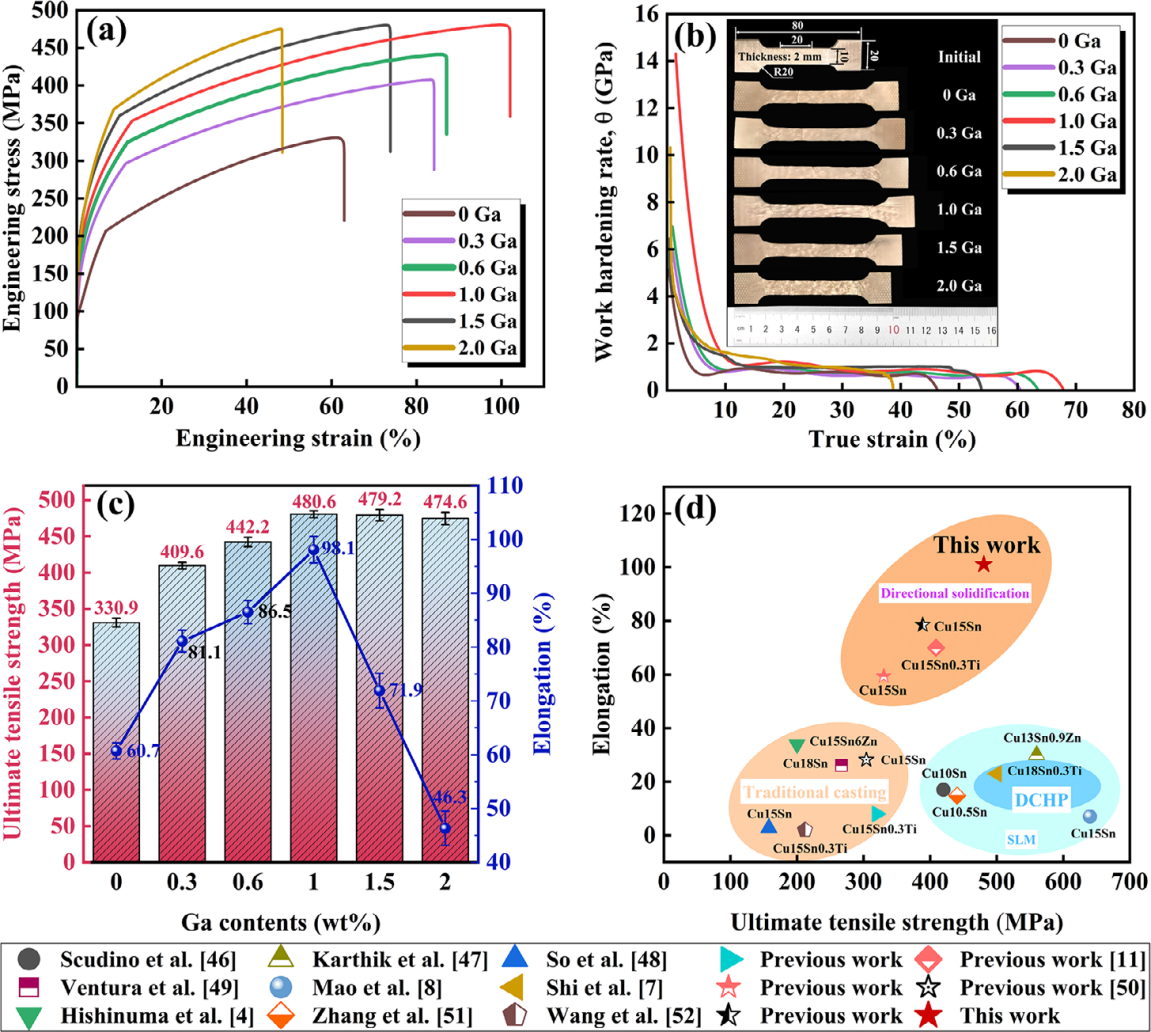
Tensile properties of alloys with different Ga contents after solution treatment. (a) Engineering stress–strain curves, (b) work‐hardening rate and true strain curves (inset denotes sample size), (c) relationship between UTS and elongation of alloys with different Ga contents, and (d) comparison of UTS and elongation of Cu–Sn alloys with similar compositions.

No obvious relationship exists between the UTS and elongation of the as‐cast alloy and Ga content. The maximum elongation of the alloy is approximately 3%, and the UTS does not exceed 300 MPa. The composition of the as‐cast alloy is extremely inhomogeneous and dominates the tensile deformation behavior of the alloy. The alloy breaks prematurely at a higher work‐hardening rate, making the relationship between mechanical properties and Ga content unclear. Therefore, the mechanical properties of the cast alloys are not discussed in depth. Figure 5 shows the tensile properties of the alloy after solid‐solution treatment. After solid‐solution treatment, the mechanical properties of the alloy are greatly improved. Specifically, as Ga addition increases, the tensile strength and elongation of the alloy first increase, reaching peak values ​​of 480.6 ± 7.2 MPa and 98.1 ± 2.7%, respectively, at a Ga content of 1.0 wt.%. As Ga addition continues to increase, the tensile strength remains constant, whereas elongation decreases rapidly. At a Ga content of 2.0 wt.%, the elongation is even lower than that of the sample without Ga addition. Previous studies have shown that the δ phase—as a preferred site for crack nucleation—severely impairs the ductility of Cu–Sn alloys. Analysis of the microstructure–mechanical property relationship of Cu–Sn alloys with different Sn contents revealed that the elongation of the alloy is inversely proportional to the volume fraction of the δ phase [10]. This indicates that the δ phase mainly affects the fracture behavior of the alloy when the Ga content exceeds 1.0 wt.% and does not contribute to the synergistic improvement of the strength and ductility of the alloy.

Figure 5b shows work‐hardening rate (*θ*) curves for different alloys with strain. In the Ga content range from 0–1.0 wt.%, the work‐hardening rate of the alloy in the initial strain stage increases with increasing Ga content. When the Ga content is 1.0 wt.%, the work‐hardening ability of the alloy remains stable for a long time without obvious strain localization. This high ductility is mainly attributed to an excellent work‐hardening ability. *θ* is essentially a manifestation of the macroscopic mechanical properties of the material. Here, the difference in *θ* arises from the evolution of the micro‐deformation mechanism dominated by the Ga content. When the Ga content exceeds 1.0 wt.%, *θ* decreases rapidly after the occurrence of minor strain and plastic instability. At this time, the stress concentration at the interface between the two phases caused by the δ phase becomes the main factor of the dominant deformation mechanism [10]. As shown in Figure 5d, compared with the undoped Ga alloy, Ga addition greatly enhances the strength–ductility of the alloy. This study therefore achieves a synergistic enhancement of the strength–ductility of the Cu–Sn alloy in contrast to the high strength obtained by the SLM method [4, 7, 8, 11, 42, 43, 44, 45, 46, 47, 48].

### 2.4 Deformation Microstructures

**FIGURE 6:**
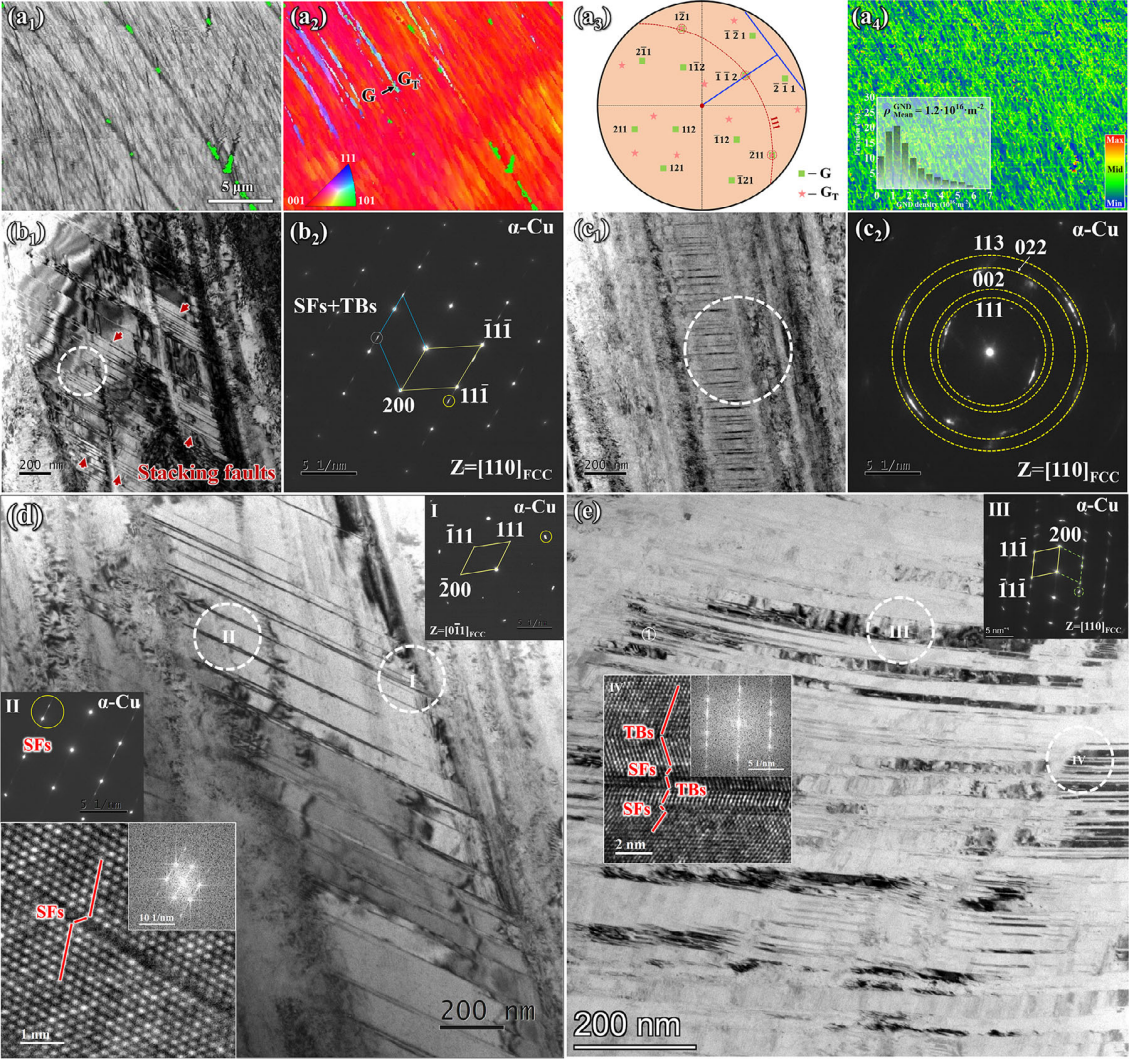
Deformation structure near the fracture of the alloy with a Ga content of 1.0 wt.%. (a) EBSD result, (b) and (c) bright field images, (d) and (e) HRTEM images.

Figure 6 shows the deformation structure observed near the fracture surface of the Cu–15Sn–1Ga alloy after tensile fracture. Figure 6a_1_,a_2_ show the BC plot and IPF results, respectively, in the electron backscattered diffraction (EBSD) mode. Structures with distinctly different orientations from the matrix are confirmed to be TBs, with a 60° misorientation angle between the TBs and matrix. The pole figure reconstruction results show three coincident points in the {111} pole figure, corresponding to the TBs and matrix (dotted circles in Figure 6a_3_), indicating that the matrix and TBs exhibit a {111}<112> twinning orientation. The trace method indicates that a (111)[$\bar{1}\bar{1}$2] orientation relationship is formed. The KAM plot indicates that the geometrically required dislocation density (GND) in the fracture microstructure is approximately 1.2 × 10^16^ m^−2^ (inset of Figure 6a_4_).

Figure 6b shows the deformed structure observed in the transmission electron microscope (TEM) mode. Figure 6b1 shows numerous dislocations entangled within the α‐Cu matrix, along with several planar defects. The tailing structure along the <111> direction, as shown in Figure 6b_2_, is a typical characteristic of SFs. Figure 6c_1_ also reveals numerous intertwining SFs with different orientations, indicating that at least two planar fault slip systems were activated. Figure 6c_2_ shows that these intertwining SFs divide the matrix into multiple nanograins, exhibiting polycrystalline diffraction patterns. Further analysis of the deformed structure is shown in Figures 6d,e. The high‐resolution TEM (HRTEM) results indicate that the numerous planar defects in Figure 6d are primarily relatively thin SFs approximately three atomic layers thick. The SAED results shown in the inset of Figure 6e, confirm that the curved structures are TBs, with evidence of SFs near the TBs. In summary, the deformed structure of the Cu–15Sn–1Ga alloy after severe plastic deformation is primarily characterized by a high density of dislocations and SFs, with a complex localized structure of TBs and SFs. SFE of pure Cu is generally reported to be relatively low at approximately 45 mJ/m^2^ [49]. Previous studies have shown that Sn addition significantly reduces the SFE of Cu–Sn alloys [10]. The deformation mechanism of the Cu–15Sn–1Ga alloy is consistent with its excellent work‐hardening ability (Figure 5b) but is fundamentally different from the twinning‐based deformation mechanism commonly found in low‐SFE alloys. This indicates that Ga doping has a significant impact on the strengthening mechanism of the alloy [50].

### 2.5 Strength–Ductility Mechanism of Ga‐Doped Cu–Sn Alloy

#### 2.5.1 Twinning Ability of Cu–Sn Alloys

**FIGURE 7:**
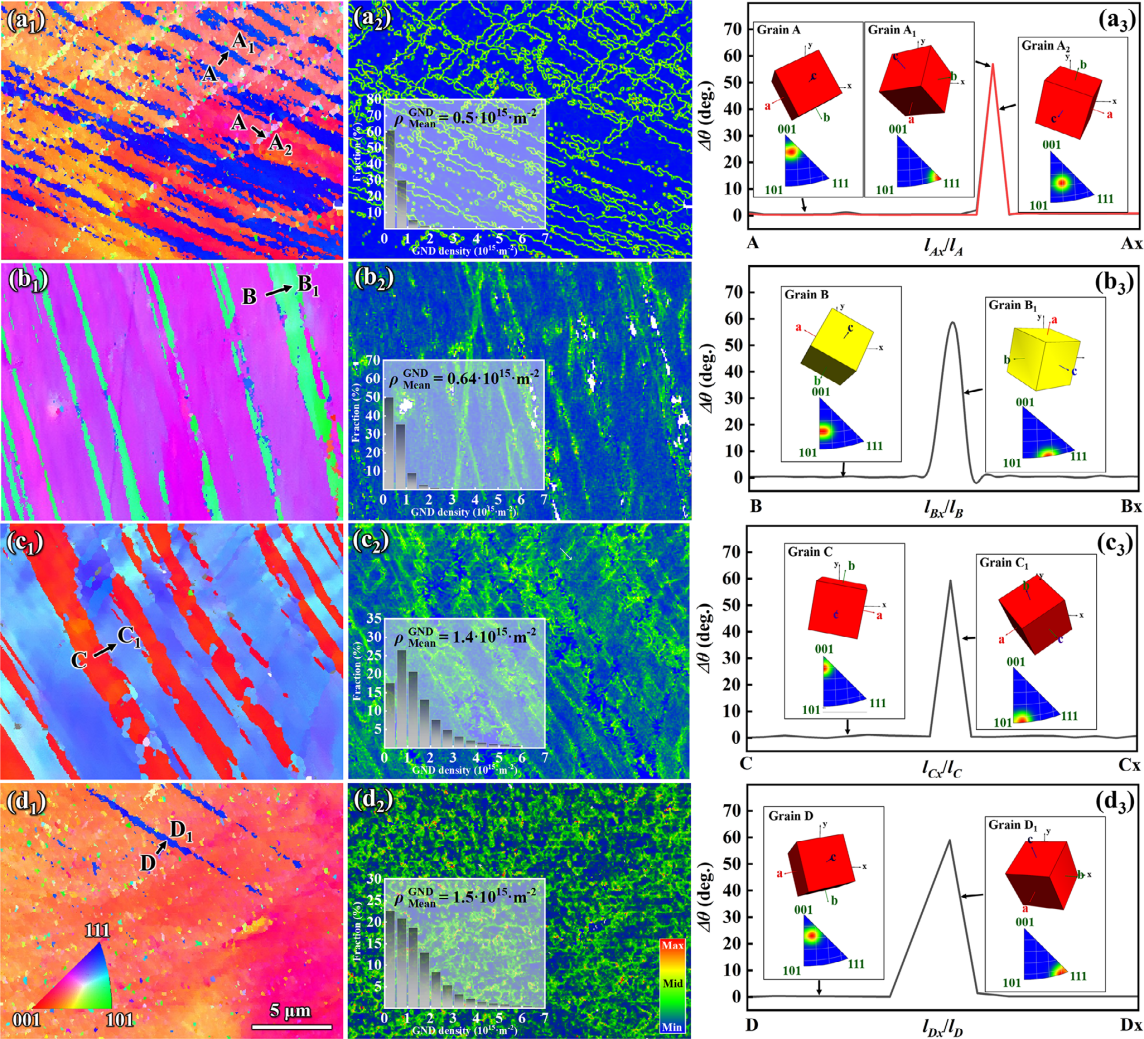
EBSD characterization of the tensile sample with different Ga contents: (a) Undoped, (b) 0.3 wt.%, (c) 1.0 wt.%, and (d) 2.0 wt.%.

**FIGURE 8:**
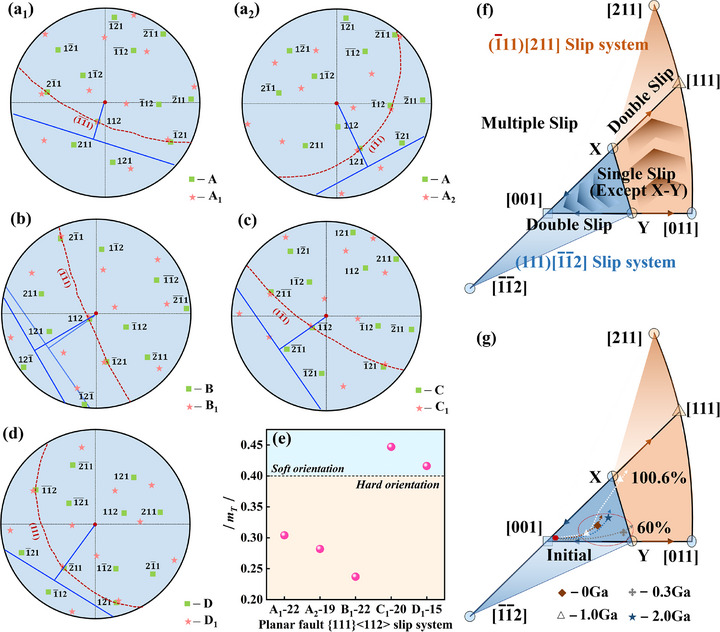
(a–d) Reconstruction of the pole figures of the matrix and TBs for alloys with different Ga contents. (e) Schmidt factor distribution of the activated planar fault slip system, (f) Lattice rotation process of {111}<112> in a standard stereographic triangle. (g) Orientation of different samples in a standard stereographic triangle.

In summary, the interaction between dislocations, SFs, and TBs is the main deformation mechanism that determines the mechanical behavior of Ga‐doped alloys, and the effect of Ga on the alloy strengthening mechanism needs to be further elucidated. Four groups of alloys with different Ga contents (0, 0.3, 1.0, and 2.0 wt.%) were designed and stretched to 60% of the rated elongation. Figure 7 shows the EBSD characterization results for the tensile sample. Figure 7a shows the IPF map, Figure 7b shows the corresponding GND statistical results, and Figure 7c shows the orientation relationship between TBs and the matrix. The results show that the orientation difference angle between all TBs and the matrix is 60°, which indicates that all TBs were formed by the activation of the {111}<112> slip system. Figure 8f,g show the initial and post‐deformation orientations of the matrix, respectively. The orientation shift path indicates that after tensile deformation, the matrix originally oriented near <001> shifts toward a <111> orientation. This is strikingly consistent with recent studies reporting that the deformation twinning in coarse‐grained Cu alloys under high‐stress conditions is largely dependent on their orientation relative to the loading direction. Under tensile loading, twinning only occurs in grains oriented in the <111> direction, where dislocation slip is possible [51, 52].

There is a close relationship between the Schmidt factor and the activity of the slip system, indicating the ease with which the slip system can be activated. For the FCC alloy, the formation of SFs and TBs of *a*/6<112> partial dislocations is dependent on the activation of the {111}<110> slip system. Therefore, the formation of surface defects requires both the {111}<112> and {111}<110> slip systems to be considered. First, the orientation distribution along the loading direction of the initial sample and that with a deformation of 60% was calculated using Equation (2) [52].

(2) $$\begin{array}{c} \left[\begin{array}{ccc} u & r & h\\ v & s & k\\ w & t & l \end{array}\right]=\left[\begin{array}{ccc} cos\varphi_{1}cos\varphi 2-sin\varphi_{1}sin\varphi_{2}cos\emptyset & sin\varphi_{1}cos\varphi_{2}+cos\varphi_{1}sin\varphi_{2}cos\emptyset & sin\varphi_{2}sin\emptyset \\ -cos\varphi_{1}sin\varphi_{2}-sin\varphi_{1}cos\varphi_{2}cos\emptyset & -sin\varphi_{1}sin\varphi_{2}+cos\varphi_{1}cos\varphi_{2}cos\emptyset & cos\varphi_{2}sin\emptyset \\ sin\varphi_{1}sin\emptyset & -cos\varphi_{1}sin\emptyset & cos\emptyset \end{array}\right] \end{array}$$

The crystal orientation is [*h* *k* *l*] = [*sin*φ_2_ *sin*∅ *cos*φ_2_ *sin*∅ *cos*∅]. Table 2 presents the statistical results for sample orientation. For the initial sample prepared by directional solidification, the exact orientation was [0.23 0.52 9.98], close to [001], with a deviation of 3.27°, showing the strongly preferred orientation of the FCC structure. In contrast, the orientations of the four modified alloys were close to [016], [319], [216], and [158]. The Schmidt factor was calculated using Equation (3) [52].

(3) $$m=\frac{F\cdot b}{\left|F\right|\cdot \left|b\right|}\cdot \frac{F\cdot n}{\left|F\right|\cdot \left|n\right|}$$

where *F* denotes the direction of the applied force, *b* denotes the slip direction of the slip system, and *n* is the normal vector of the slip plane of the slip system. *m_S_* and *m_T_* for the octahedral dislocation slip and planar fault slip systems were also calculated and are listed in Tables 3 and 4, respectively. In particular, when the Schmidt factor is greater than 0.4, the slip system is in a soft orientation and is easily activated; conversely, it is difficult to activate [51, 52]. Table 5 lists the order of slip systems that are theoretically easy to activate for different samples according to the Schmidt factor. The results show that as deformation progresses, the orientation shifts, and the number of activatable slip systems decreases accordingly. However, at least two {111}<110> and {111}<112> slip systems were still activated.

**TABLE 2:** Averaged Euler angles and orientation of different samples.

Samples		Average all Euler angles			Orientation	Approaching orientation	Deviation
		φ_1_	Ф	φ_2_			
Initial		132.15	3.27	23.3	[0.23 0.52 9.98]	[001]	3.27
ε = 60%	0 Ga	335.25	10.67	17.18	[0.55 1.77 9.83]	[016]	3.22
	0.3 Ga	138.19	18.96	66.23	[2.97 1.31 9.46]	[319]	1.80
	1.0 Ga	126.79	20.1	65.86	[3.14 1.41 9.39]	[216]	0.91
	2.0 Ga	142.78	31.2	7.91	[0.71 5.13 8.55]	[158]	2.22

**TABLE 3:** Schmid factors of octahedral {111}<110> slip systems for alloys with different Ga contents.

Slip system No.	Slip plane	Burgers vector	Schmid factor				
			Initial	0 Ga	0.3 Ga	1.0 Ga	2.0 Ga
1	(111)	[$\bar{1}$01]	0.428	0.460	0.364	0.356	0.461
2	(111)	[$0\bar{1}$1]	0.415	0.400	0.457	0.454	0.201
3	(111)	[$\bar{1}$10]	0.013	0.061	−0.093	−0.099	0.260
4	($\bar{1}$11)	[$0\bar{1}$1]	0.397	0.363	0.259	0.250	0.181
5	($\bar{1}$11)	[101]	0.429	0.468	0.396	0.392	0.491
6	($\bar{1}$11)	[110]	0.031	0.104	0.136	0.142	0.309
7	(1$\bar{1}$1)	[$\bar{1}$01]	0.386	0.326	0.294	0.284	0.1324
8	(1$\bar{1}$1)	[011]	0.415	0.407	0.489	0.490	0.231
9	(1$\bar{1}$1)	[110]	0.030	0.081	0.194	0.206	0.099
10	(11$\bar{1}$)	[011]	−0.396	−0.356	−0.227	−0.214	−0.151
11	(11$\bar{1}$)	[101]	−0.385	−0.318	−0.263	−0.248	−0.103
12	(11$\bar{1}$)	[$\bar{1}$10]	−0.011	−0.037	0.035	0.034	−0.049

**TABLE 4:** Schmid factors of Planar fault {111}<112> slip systems for alloys with different Ga contents.

Slip system No.	Slip plane	Burgers vector	Schmid factor				
			Initial	0 Ga	0.3 Ga	1.0 Ga	2.0 Ga
13	(111)	[11$\bar{2}$]	−0.486	−0.496	−0.474	−0.468	−0.382
14	(111)	[$1\bar{2}$1]	0.232	0.196	0.318	0.319	−0.034
15	(111)	[$\bar{2}1$1]	0.254	0.301	0.156	0.149	0.416
16	($\bar{1}$11)	[211]	0.266	0.330	0.307	0.308	0.463
17	($\bar{1}$11)	[$\bar{1}1\bar{2}$]	−0.477	−0.480	−0.378	−0.370	−0.388
18	($\bar{1}$11)	[$\bar{1}\bar{2}$1]	0.211	0.150	0.071	0.062	−0.074
19	(1$\bar{1}$1)	[121]	0.257	0.282	0.395	0.402	0.190
20	(1$\bar{1}$1)	[$1\bar{1}\bar{2}$]	−0.463	−0.423	−0.452	−0.447	−0.210
21	(1$\bar{1}$1)	[$\bar{2}\bar{1}$1]	0.206	0.141	0.058	0.045	0.019
22	(11$\bar{1}$)	[112]	−0.330	−0.304	−0.237	−0.225	−0.130
23	(11$\bar{1}$)	[$\bar{2}$1$\bar{1}$]	0.354	0.274	0.235	0.220	0.070
24	(11$\bar{1}$)	[1$\bar{2}\bar{1}$]	0.235	0.227	0.111	0.104	0.116

**TABLE 5:** Slip systems that could be activated for alloys with different Ga contents.

Samples		Octahedral {111}<110> slip systems	Planar fault {111}<112> slip systems
Initial		5, 1, 2, 8	13, 17, 20
ε = 60 %	0 Ga	5, 1, 8, 2	13, 17, 20
	0.3 Ga	8, 2	13, 20
	1.0 Ga	8, 2	13, 20, 19
	2.0 Ga	5, 1	16, 15

Figure 8a–d show the reconstructions of all TBs and the matrix {111} pole figures in Figure 7. Among them, the two slip systems activated by the undoped Ga alloy are ($\bar{1}\bar{1}$1)[112] and (1$\bar{1}$1)[121], Cu–15Sn–0.3Ga corresponds to ($\bar{1}\bar{1}$1)[112], Cu–15Sn–1Ga corresponds to ($1\bar{1}$1)[11$\bar{2}$], and Cu–15Sn–2Ga corresponds to (111)[$\bar{2}$11], which correspond to slip systems 22, 19, 22, 20, and 15 in the theoretical calculation results presented in Table 4, respectively. Figure 8e shows the distribution of soft and hard orientations for the five planar fault {111}<112> slip systems. These results, combined with the calculated results presented in Table 5, clearly indicate that the theoretically easy‐to‐activate {111}<112> slip systems in the undoped Ga and Cu–15Sn–0.3Ga alloys were not activated, whereas slip systems with hard orientations were activated. A stark contrast occurred in the Cu–15Sn–1Ga and Cu–15Sn–2Ga alloys, where all activated slip systems had soft orientations. With increasing Ga content, the activation of the {111}<112> slip system—which dominates twinning behavior in the alloys—becomes increasingly difficult, suggesting that Ga addition inhibits the twinning ability of the alloy. The effect of Ga on the twinning ability of the alloy is also reflected in the number of activated {111}<112> slip systems. As shown in Figure 7, the undoped Ga alloy activates at least two slip systems that were extremely difficult to activate, resisting plastic deformation through a deformation mechanism involving multiple planar fault interactions. However, with the addition of Ga, only the more easily activated {111}<112> slip system was activated, indicating that Ga addition suppresses the twinning ability of the alloy [53].

#### 2.5.2 From Dislocations and SFs to TBs and Their Intersection

**FIGURE 9:**
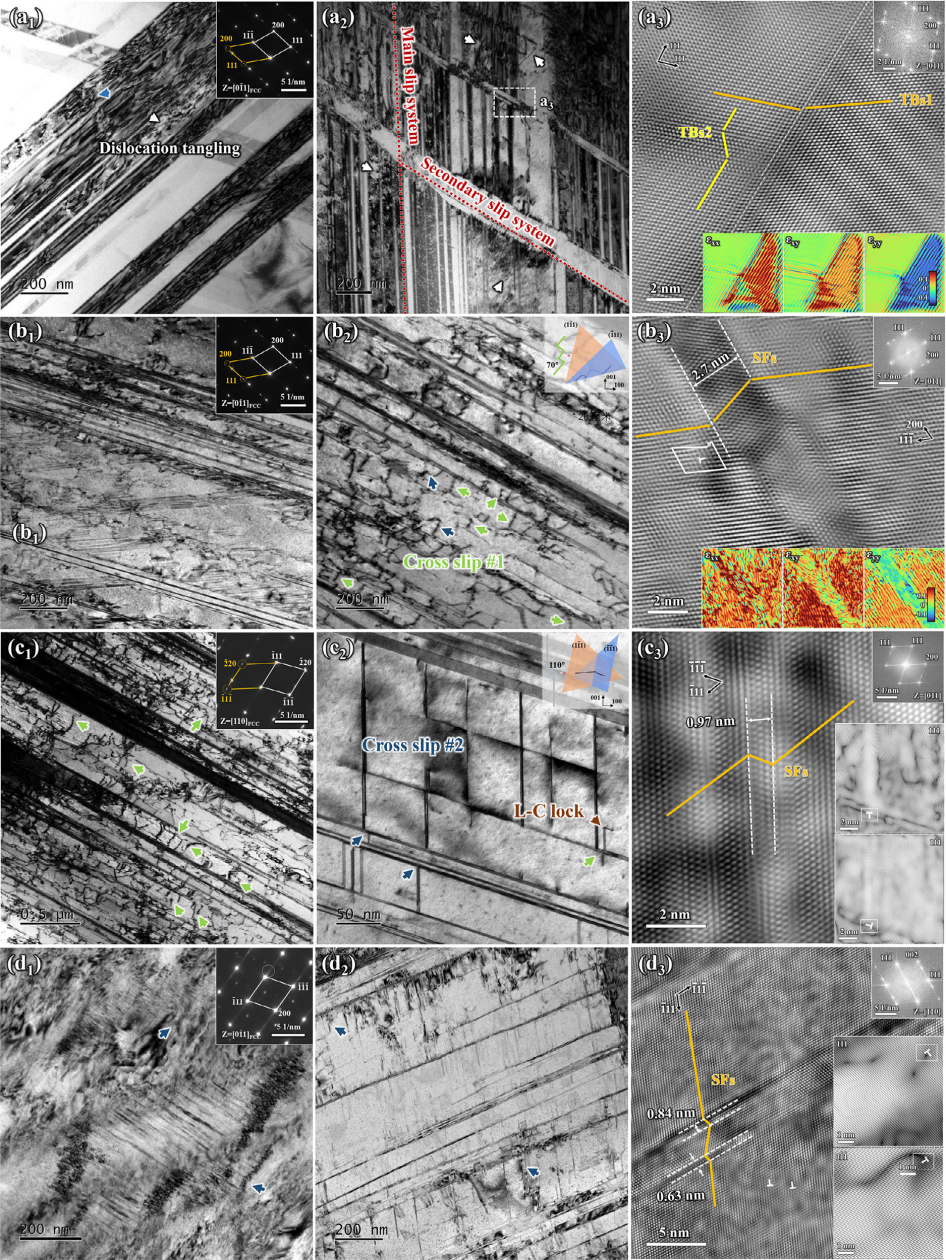
TEM and HRTEM images of the tensile sample with different Ga contents: (a) undoped Ga, (b) 0.3 wt.%, (c) 1.0 wt.%, and (d) 2.0 wt.%.

As previously mentioned, the twinning ability of the alloy decreased with increasing Ga content, whereas the GND results showed the opposite trend (Figure 7a2–d_2_), indicating that the ability of the alloy to slip and accumulate dislocations improved. The microstructure of the alloy at the diameter reduction point under TEM mode is shown in Figure 9. The undoped Ga alloy exhibits a high density of TBs. The interactive structure of the TBs is similar to that observed in EBSD, where at least two planar fault {111}<112> slip systems are activated. Unlike the undoped Ga alloy, the SAED pattern of the Cu–15Sn–0.3Ga alloy exhibits typical features of the SF structure, a phenomenon also observed in other Ga‐containing alloys. The results of the strain distribution analysis of the HRTEM images are shown in Figure 9a3,b_3_. The strain distributions of the TBs in the two directions of the undoped Ga alloy is not consistent, and the higher strain is mainly distributed on the side of the TBs formed by the activation of the primary slip system. Activation of the secondary slip system has been shown to relieve the local stress concentration caused by primary TBs. Therefore, the strain state around the secondary twins is opposite to that of the initial twins, which are tensile and compressive stresses, respectively. In contrast to the case of TBs, a more uniform strain is generated around the SFs, which is related to the fact that the scale of SFs consists of several atomic layers, whereas TBs are symmetrical arrangements of atoms on two planes. The latest research suggests that the two structures contribute differently to the mechanical properties of the alloy. TBs are prone to cause local stress concentrations and contribute more to strength, whereas SFs are more conducive to ductility [54].

Both TBs and SFs are associated with the activation of the planar fault {111}<112> slip system, which is closely related to the activation of the {111}<112> slip system. SFs are generated by full dislocation decomposition, and their appearance is considered a precursor to the formation of TBs. The *a*/2<110> dislocation decomposes into two *a*/6<112> Shockley partial dislocations. The decomposition reaction is as follows:

(4) $$\frac{a}{2}\left[110\right]\to \frac{a}{6}\left[211\right]+\frac{a}{6}\left[12\bar{1}\right]+CSF$$

*a*/6[211]—as the leading partial dislocation—is located at the forefront of the movement and is the first to leave the full dislocation from the dislocation accumulation point. A complex stacking fault (CSF) moves closely behind it, accompanied by the trailing partial dislocation *a*/6$\left[12\bar{1}\right]$ [55]. The CSF is generated by twin partial dislocations during the initiation of TBs. As more *a*/2<110> dislocations decompose in the same manner on the adjacent {111} planes, the formed multilayer CSF transforms into microtwins through short‐range diffusion of the reordering mechanism. Continuous reactions of the same type lead to the thickening and growth of TBs [52]. Once can reasonably speculate that the full dislocation in the undoped Ga alloy can fully react with ease according to Equation (4). At this time, the number of *a*/2<110> dislocations decreases, corresponding to an increase in the volume fraction of the planar fault. This phenomenon differs for Ga‐containing alloys. The ease of dislocation decomposition can be determined by measuring the distance of incomplete dislocation decomposition. The dislocation decomposition distance of the Cu–15Sn–0.3Ga alloy is approximately 2.7 nm, that of the Cu–15Sn–1Ga alloy is 0.97 nm, and that of the Cu–15Sn–2Ga alloy is 0.84 nm. SFE is typically used to describe the difficulty of dislocation decomposition. A lower SFE makes it easier for full dislocations to decompose, thereby forming thicker SF sheets, indicating that the SFE of the alloy increases with increasing Ga content [56]. Meanwhile, the neatly arranged dislocation entanglements observed at the TBs in the undoped Ga alloy are characteristic of plane‐slip (white arrows in Figure 9a1,a_2_). The oscillating dislocation lines observed in the brightfield phase are considered to be the result of the cross‐slip of screw dislocations on multiple {111} planes (green and blue arrows in Figure 9b_2_). These two types of cross‐slip may represent different combinations of the {111} planes, and their morphologies are shown in the insets of Figure 9b2,c_2_, respectively. The promoting effect of Ga addition on cross‐slip is also closely related to the influence of SFE. Wen et al. reported that element doping improves the SFE of medium‐entropy alloys and promotes the transformation of dislocations from plane‐slip to cross‐slip, thereby improving the properties of the alloys [57]. Cross‐slip requires dislocation transfer from the original slip plane to an adjacent slip plane. In this process, the energy difference between dislocations in two different local environments must be overcome. Because the distance between partial dislocations of Ga‐containing alloys is small, the constraints between the partial dislocations are weak, and they can easily break away from the original plane, thereby promoting cross‐slip [58, 59]. Furthermore, in the HRTEM morphologies of the Cu–15Sn–1Ga and Cu–15Sn–2Ga alloys, dislocations can be observed at the overlapping positions of the two {111} planes (Figure 9c3,d_3_), which also indicates the occurrence of cross‐slip.

The effect of Ga on the slip capacity can be explained by the actuated slip system. For the undoped Ga alloy, four activated {111}<110> octahedral slip systems (5, 1, 8, and 2) and two actually activated {111}<112> plane fault slip systems (19 and 22) exist. These {111}<112> plane fault slip systems can be generated by the decomposition of an octahedral {111}<110> slip system. Therefore, the formation of plane faults in undoped Ga alloys can be described as follows:

(5) $$\begin{array}{c} \frac{\frac{a}{2}{\left[011\right]}_{on\left(1\bar{1}1\right)}}{No.\;8\;slip\;system}\to \frac{\frac{a}{6}{\left[121\right]}_{on\left(1\bar{1}1\right)}+CSF}{No.19\;slip\;system}+\frac{\frac{a}{6}{\left[1\bar{12}\right]}_{on\left(1\bar{1}1\right)}}{No.\;20\;slip\;system} \end{array}$$

The 22nd slip system that induces a twinning interaction structure is also activated in the Cu–15Sn–0.3Ga alloy. The generation of the twinning interaction structure may be derived from the activation of the 10th or 11th octahedral slip system in a non‐soft orientation, which means that undoped Ga and Cu–15Sn–0.3Ga alloys have limited slip capabilities. In the Cu–15Sn–1Ga and Cu–15Sn–2Ga alloys, the 15th and 20th slip systems are derived from the activation of the first and eighth slip systems, respectively, which are also in a soft orientation. This indicates that the Cu–15Sn–1Ga and Cu–15Sn‐2Ga alloys have stronger slip abilities. The 20th slip system decomposes from the eighth slip system, and the decomposition reaction is the same as that in Equation (5), whereas the activation of the 15th slip system is derived from the following reaction:

(6) $$\begin{array}{c} \frac{\frac{a}{2}{\left[\bar{1}01\right]}_{on\left(111\right)}}{No.\;1\;slip\;system}\to \frac{\frac{a}{6}{\left[\bar{2}11\right]}_{on\left(111\right)}+CSF}{No.\;15\;slip\;system}+\frac{\frac{a}{6}{\left[\bar{1}\bar{1}2\right]}_{on\left(1\bar{1}1\right)}}{No.\;13\;slip\;system} \end{array}$$

#### 2.5.3 Slip and Twinning

**FIGURE 10:**
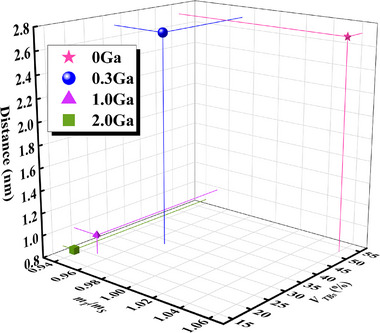
Relationship between dislocation decomposition width, *m*_T_/*m*_S_, and volume fraction of planar fault structure (*V*_TBs_) in alloys with different Ga contents.

Studies have shown that the ratio of the maximum Schmidt factor (*m_T_*) of the slip system that causes plane faults to the maximum Schmidt factor (*m_S_*) of the dislocation slip system is closely related to the tendency for slip and twinning [51]. Using *V_TBs_* to represent the volume fraction of plane faults in the alloy, the relationship between dislocation decomposition width, *m_T_*/*m_S_*, and *V_TBs_* in alloys with different Ga contents is shown in Figure 10. The results show that with increasing Ga content, the values of *V*_TBs_, *m*_T_/*m*_S_, and dislocation decomposition distance all decrease. As the Ga content increases from 0 to 2.0 wt.%, the volume fraction of the planar fault structure decreases from 52.4% to 17.7%, while *m*_T_/*m*_S_ decreases from 1.06 to 0.94. As mentioned previously, the decrease in dislocation decomposition distance with increasing Ga content indicates that slip becomes the dominant deformation mechanism. Both theoretical calculations and experimental results demonstrate that Ga addition effectively coordinates the competition between slip and twinning.

**FIGURE 11:**
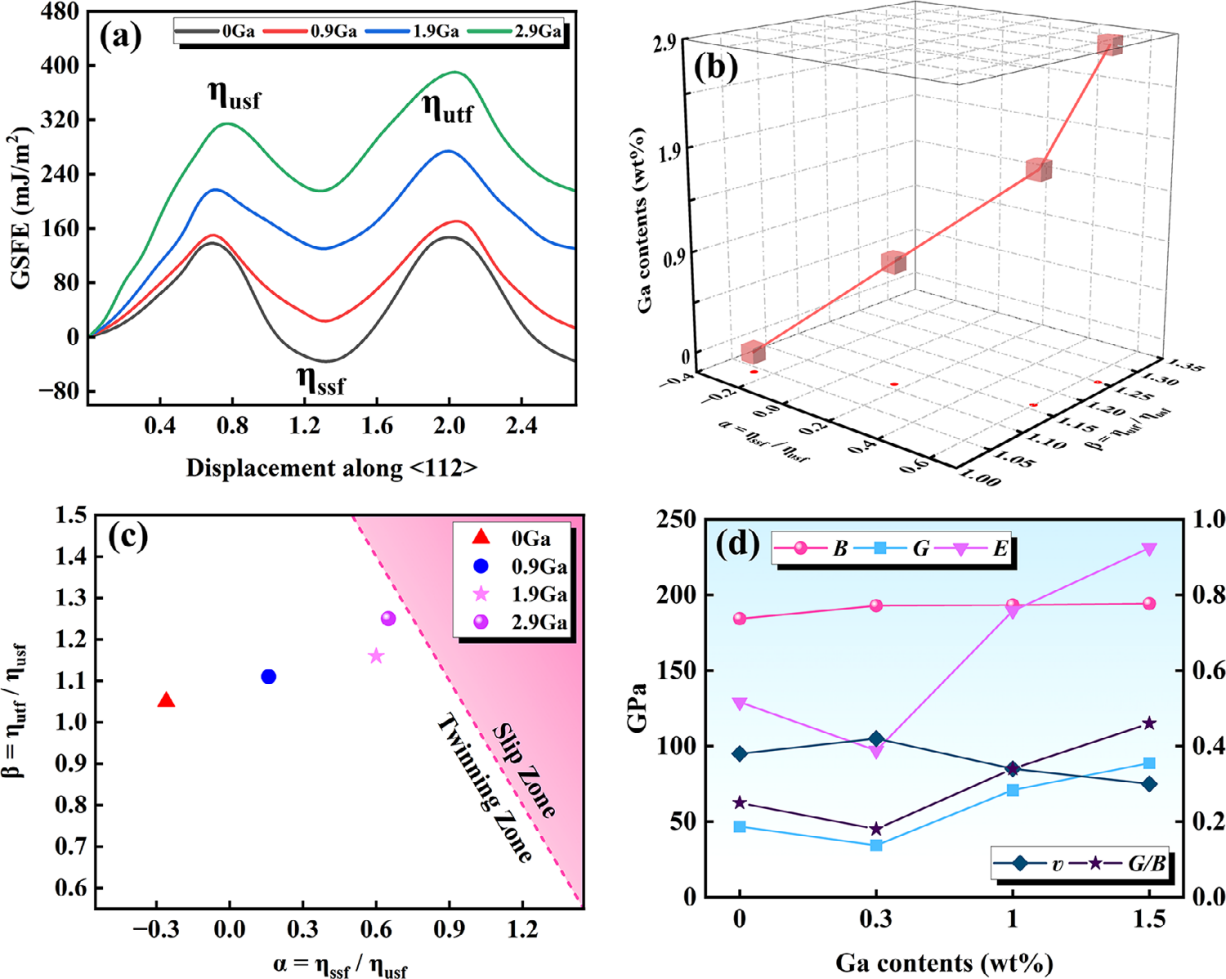
(a) GSFE results for Cu–Sn alloys with different Ga contents, (b) changes in *α* and *β* values at different Ga contents, (c) GSFE curves in *α*‐*β* coordinates, and (d) elastic constants and Poisson's ratio.

Both slip and twinning are profoundly regulated by the SFE of the alloy. By influencing the tendency of the total dislocations to decompose, SFE directly determines the competitive relationship between dislocation motion and twinning—the two plastic deformation mechanisms. The calculated SFE results for the Cu–Sn alloys with different amounts of Ga are shown in Figure 11a. The stable stacking fault energy (*η_ssf_*), unstable stacking fault energy (*η_usf_*), and unstable twinning energy (*η_utf_*) all increase with increasing Ga content. The two parameters, *α* = *η_ssf_* /*η_usf_* and *β* = *η_utf_* /*η_usf_*, are usually introduced to quantify the competitive relationship between the slip and twinning mechanisms of the alloy during plastic deformation (Figure 11b). Specifically, this competitive relationship can be described by the following critical criterion: *α* + *β* = 2. As shown in Figure 11c, the coordinate plane established according to this criterion is divided into twinning and slip zones [55, 60]. Note that the simulation results are surprisingly consistent with the experimental results. As the Ga content increases, the alloy gradually falls from the twinning zone to the slip zone, indicating that Ga addition causes the twinning ability of the alloy to decrease, and slip becomes the deformation mechanism, gradually replacing twinning. The calculated elastic properties of the α‐Cu matrix, including bulk modulus (*B*), shear modulus (*G*), elastic modulus (*E*), Poisson's ratio (*ν*), and *G/B*, are shown in Figure 11d. The results show that after adding 0.3 wt.% Ga, *B* is higher than pure α‐Cu, *G* and *E* are lower than pure α‐Cu, and *ν* is slightly higher than pure α‐Cu. As the Ga content increases from 0.3 to 1.5 wt.%, *B*, *G* and *E* show linear increases, whereas *ν* gradually decreases. However, *G*/*B* shows a completely opposite change to that of *ν*, that is, *G/B* decreases when the Ga content is less than 0.3 wt.%, and then gradually increases with the increase in Ga content. *E* represents the ability of the system to resist elastic deformation, *B* represents the ability of the material to resist fracture, and *G* represents the ability of the material to resist shear strain. A *G/B* of 0.57 is the transition point between the brittleness and toughness of metal materials. When *G/B* is greater than 0.57, the material is brittle material, whereas below, it is ductile. As the Ga content increases, the *G/B* value first decreases, and then rapidly increases to 0.46, indicating that the ductility of the alloy gradually decreases. Moreover, the closer Poisson's ratio of the material is to 1/3, the better the ductility. Poisson's ratio of the four‐component alloys first approaches and then moves away from the limit of 1/3 as the Ga content increases [61]. One can infer that the alloy reaches the plastic limit when the Ga content is 1.0 wt.%. Note that the calculated results are surprisingly consistent with the test results of the mechanical properties of the alloy. That is, the three‐dimensional numerical simulation, theoretical calculation, and experimental verification all confirm that with the addition of Ga, the deformation mechanism changes from twinning to slip. This balance between plane faults and slip causes the alloy to achieve a synergistic enhancement in strength–ductility.

### 2.6 Effect of Ga on the Superconducting Properties of Nb_3_Sn

**FIGURE 12:**
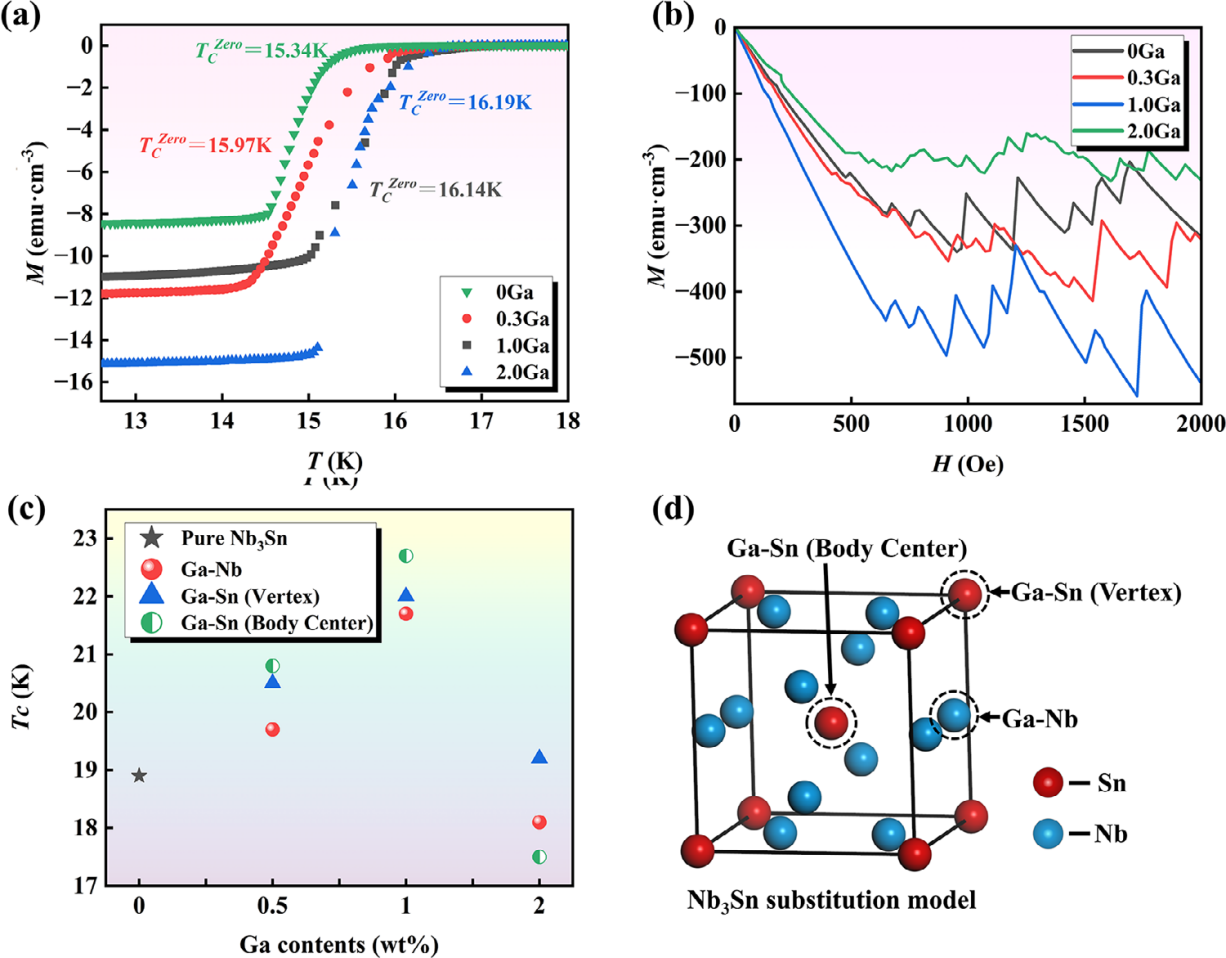
Test and calculation results of Nb_3_Sn superconducting properties: (a) M–T curves, (b) M–H curves, (c) *T_c_* calculated results, (d) schematic of different substitution positions of Ga atoms.

In light of previous research on the beneficial effects of Ga doping of Nb_3_Sn, this study is the first to demonstrate a significant improvement in the mechanical properties of Cu–Sn alloys by Ga doping [23, 28, 29]. Therefore, further verification of the effects of the proposed doping method and amount on the superconducting properties of the Nb_3_Sn phase is necessary. Figure 12a shows the relationship between magnetization intensity and temperature (M–T) curves for Nb_3_Sn films with different Ga doping levels, and Figure 12b shows the relationship between magnetization intensity and magnetic field intensity (M–H) curves. The M–T test results show that compared with undoped Ga, trace Ga doping significantly improves the *T*_c_ value, and the *T*_c_ value gradually increases with increasing Ga content. The *T*_c_ value is the lowest for the undoped Ga sample at 15.34 K, compared to that of the Cu–15Sn–0.3Ga sample at 15.97 K. The *T*_c_ values reach the peak at 1.0 and 2.0 wt.% Ga doping and are relatively close at this composition at 16.14 and 16.19 K, respectively. When the Ga doping amount exceeds 1.0 wt.%, the *T*_c_ values are relatively close, which may be related to the saturation of the Nb and Sn sites in the Nb_3_Sn phase by Ga substitution. Studies have confirmed that the mechanism by which Ga improves superconducting properties differs from that of other doping elements. Ga can directly enter the Nb_3_Sn phase and randomly replace Nb or Sn atoms to form (Nb, Ga)_3_Sn or Nb_3_(Sn, Ga) phases, thereby improving superconducting properties. Similar to the atomic substitution mechanism in substitutional solid solutions, when the concentration of Ga in the Nb_3_Sn phase reaches a critical value, the *T*_c_ value does not change significantly as the Ga content continues to increase [23, 62]. The M–H test results show that the lower critical magnetic field strength (*H*_c1_) of the Nb_3_Sn superconducting films with different compositions is almost unaffected by the Ga content. The *H*_c1_ value fluctuates around 647.7 Oe for all Ga contents. *H*_c1_ is the intrinsic superconducting property of superconducting materials, representing the critical magnetic field strength when the superconductor transitions from the Meissner state (perfect diamagnetism) to a mixed state. The above results indicate that Ga doping does not significantly change the limiting operating magnetic field strength of the Nb_3_Sn superconductor. Comparing the *M* values for the same *H*_c1_ reveals that the Nb_3_Sn sample prepared from the Cu–15Sn–1Ga alloy has the highest magnetization, whereas that at Ga contents of 0 and 0.3 wt.% is lower. Notably, the lower magnetization at a Ga content of 2.0 wt.% may be related to the intrinsic property changes caused by the structural alteration of the Nb_3_Sn phase owing to excessive Ga addition. According to the Bean critical state model [63], in the mixed state—where *H* > *H*_c1_—the larger |*M*| is, the larger the area of the geometric region formed by the M–H curve and coordinate axis, and the material can sense and maintain a stronger shielded superconducting current. That is, |*M*| is directly proportional to *J*_c_, which indicates that 1.0 wt.% Ga doping can synergistically improve the *T*_c_ and *J*_c_ of Nb_3_Sn superconductors. Because *T*_c_ is an intrinsic parameter of the Nb_3_Sn phase and the Nb_3_Sn phase has a specific crystal structure and atomic occupancy, its superconducting properties are difficult to change through the preparation process or environment. Therefore, the increase in *T*_c_ owing to elemental doping is not revolutionary (approximately 0.85 K), which is expected. Compared with the *T*_c_ value of 14–15 K obtained by Wenura et al. [62], further improvement of *T*_c_ presented in this study will have an important impact on the entire superconducting field. Second, although the *T*_c_ improvement brought about by Ga doping is gradual, it may still bring about a significant synergistic improvement in *T*_c_ and *J*_c_ in the actual high‐field magnet operating temperature range.

Within the BCS theoretical framework, the *T_c_* calculation equation proposed by McMillan [64] can explain the above experimental phenomena. The theoretical value of *T_c_* can be calculated by the following equation:

(7) $$T_{c}=\frac{\Theta_{D}}{1.45}\exp\left[-\frac{1.04\left(1+\lambda \right)}{\lambda -\mu^{*}\left(1+0.62\lambda \right)}\right]$$

where *θ_D_* is the Debye temperature:

(8) $$\Theta_{D}=\frac{h}{k}{\left(\frac{3nN_{A}\rho }{4\pi M}\right)}^{1/3}V_{m}$$

(9) $$V_{m}={\left[\frac{1}{3}\left(\frac{2}{V_{S}^{3}}+\frac{1}{V_{L}^{3}}\right)\right]}^{-1/3}$$

(10) $$V_{S}=\sqrt{\frac{G}{\rho }}$$

(11) $$V_{L}=\sqrt{\frac{\left(B+4G/3\right)}{\rho }}$$

*G* is the shear modulus:

(12) $$G=\frac{G_{V}+G_{R}}{2}$$

(13) $$G_{V}=\frac{3c_{44}+c_{11}-c_{12}}{5}$$

(14) $$G_{R}=\frac{5c_{44}\left(c_{11}-c_{12}\right)}{4c_{44}+3\left(c_{11}-c_{12}\right)}$$

B is the bulk modulus, and E is the Young's modulus:

(15) $$B=\frac{c_{11}+2c_{12}}{3}$$

(16) $$E=\frac{9GB}{G+3B}$$

and µ^*^ is the electron Coulomb repulsion potential parameter:

(17) $$\mu^{*}=\frac{0.26N\left(E_{F}\right)}{1+N\left(E_{F}\right)}$$

For the density of states at the Fermi surface *N(E_F_)* and *B*, *G* is defined by first‐principles calculations, *h* is the Planck constant, *k* is the Boltzmann constant, *N_A_* is the Avogadro constant, *n* is the unit number of atoms, *M* is the unit molecular mass, and *ρ* is the density. Based on these results, nine Nb_3_Sn substitution models were developed. Taking 0.5wr%Ga doping to replace Nb sites as the example shown in Figure 12c, where Θ_*D*_ is 318.0 K in the pure Nb_3_Sn model, *T_c_* is the reported 18.9 K, and the electroacoustic coupling coefficient *λ* calculated at 1.7 [65] is substituted into Equation (7). Regardless of whether Ga occupies the Nb or Sn sites, *T_c_* always increases first and then decreases with increasing Ga content. The three doping sites exhibit the same trend, and the increase in *T_c_* is greater when Ga occupies the Sn site. Notably, the Cu–Sn alloy doped with 1.0 wt.% Ga exhibits the best mechanical properties, and the Nb_3_Sn superconductor prepared with this Ga addition also exhibits the best superconducting properties.

These results demonstrate for the first time the synergistic strengthening effect of Ga addition on both the front‐end alloy and back‐end superconductor, and detail the doping methods and amount that can be used as a reference for production. This has important practical significance for overcoming the bottleneck that is the mechanical properties of existing Cu–Sn alloys and improving the property limits of Nb_3_Sn superconducting wires. This will significantly boost the development of controlled nuclear fusion projects, as exemplified by ITER. More importantly, the material design and strengthening concept proposed in this study—which balances both the front‐end and back‐end—will provide new insights into the development of similar doping‐strengthened metallic materials and bring new insights into solving major engineering challenges.

## 3 Conclusions

In this study, by adjusting the amount of Ga added, the strength–ductility of Cu–Sn alloys prepared by directional solidification were both enhanced. The optimal Ga content was further confirmed to significantly enhance the superconducting properties of Nb_3_Sn. The main conclusions are directly supported by the following experimental evidence:

1. Ga was mainly distributed in the dendritic trunk, forming a competitive solid solution relationship with Sn, resulting in an increase in the volume fraction of the δ phase with increasing Ga content. When the Ga doping content was 1.0 wt.%, the strength and ductility of the alloy both reached their peak values, with a UTS of 480.6 ± 7.2 MPa and an elongation of 98.1 ± 2.7%. As the Ga content continued to increase, the UTS of the alloy remained unchanged, whereas the elongation gradually decreased.
2. The deformed structure of the undoped Ga alloy was mainly composed of high‐density TBs. With increasing Ga content, the proportions of SFs and dislocations in the deformed structure increased significantly, the dislocation decomposition distance gradually decreased, and the cross‐slip phenomenon became more common, indicating that the deformation mechanism of the alloy changed from twinning to slip.
3. Ga‐doping increased the Tc of Nb3Sn from 15.34 to 16.19 K at 2.0 wt.% Ga doping. The Hc1 value was almost unaffected by the Ga content, and Nb3Sn exhibited the highest magnetization when doped with 1.0 wt.% Ga.

The main conclusions based on existing theory and calculation are as follows:

1. The formation energy of Ga atoms entering the Cu lattice was lower than that of Sn, indicating that the solid‐solution process was thermodynamically more favorable. As the Ga content increased, Sn was repelled into the interdendritic space, and the volume fraction of the δ phase increased accordingly, leading to a sharp decrease in the ductility of the alloy when Ga doping exceeded 1.0 wt.%.
2. Ga‐doping improved the SFE of the alloy, and with increasing Ga content, the deformation mechanism shifted from the twinning region to the slip region. Schmidt factor analysis showed that the difficulty in activating the slip system that dominates the planar fault increased with increasing Ga content.
3. Ga substitution at both the Nb and Sn sites increased the Tc of Nb3Sn, with the effect of Sn site substitution being more significant. Tc reached its peak at a Ga‐doping concentration of 1.0 wt.%. The Jc of Nb3Sn also improved at this doping concentration.

## 4 Experimental Methods

### 4.1 Raw Materials and Experimental Procedure

The Bridgman technique was used for directional solidification of the Cu–Sn alloy, resulting in grains that grew in a specific direction. This process was conducted in a vacuum‐based directional solidification furnace under a significant temperature gradient. The rate at which the alloy was withdrawn from the melt was carefully controlled at 100 µm/s, leading to the production of alloy rods with dimensions of Φ24 mm × 200 mm. The chemical composition of the alloy—expressed in weight percent (wt.%)—included Sn–15.0, Ga–0, and varying concentrations of 0.3, 0.6, 1.0, 1.5, and 2.0, with the remainder being (Cu–Bal). Oxygen‐free Cu, pure Sn, and pure Ga (each with purity exceeding 99.99%) were used. The alloy rods were abraded with 2000‐grit SiC sandpaper and subsequently polished to achieve a smooth surface. The rods were then subjected to a complete solution treatment at 650°C for a duration of 72 h, which were the optimal process parameters obtained from previous work [11]. Following solution treatment, the rods were cooled rapidly using water, with the cooling rate estimated to be approximately 200 K/s.

### 4.2 Microstructural Characterization

To characterize the microstructure of the alloy, the 10 mm × 10 mm surface of the Cu–Sn alloy was ground, polished, and cleaned. The alloy specimens were then electrolytically etched with a solution consisting of 20 mL H_3_PO_4_ and 80 mL anhydrous ethanol at a voltage of 5 V and a current of 1 A. A Phenomenon Pure + field desktop scanning electron microscope (SEM) was used to observe the microstructures of the alloy rods. Their composition was analyzed using a JEC‐300FC EPMA. XRD was conducted on a Rigaku X‐Ray diffractometer, with Cu–Ka radiation used to confirm the phases present in the alloy. The scanning angle was 10–90°, and the scanning rate was 5°/min. The tube voltage and current were 45 kV and 200 mA, respectively. The precise microstructure information for the cross‐sections along the directional solidification direction of all samples before and after tensile testing was examined using a Zeiss Merlin Compact SEM fitted with an EBSD detector, and the characterization area of the sample after tensile testing was selected near the fracture. EBSD data were obtained from a region of 20 µm × 20 µm, the EBSD scan step size was 0.05 µm, and EBSD data were mainly analyzed using HKL Channel 5 software. The GND densities of the samples were calculated using ATEX software. The phase and deformation microstructures were observed and analyzed using a Talos F200X TEM at an accelerating voltage of 200 kV, and the composition was analyzed using super EDS. TEM film specimens with diameters of 3 mm and thicknesses of approximately 40 µm were conducted in the vicinity of the fracture of the alloy after all tensile testing. The specimens were then thinned using a double‐jet thinning device. The size and area fraction of the phases in the alloy were determined using Image‐Pro Plus software. To ensure the accuracy of the measurements, three photographs were taken to measure the microstructure at different locations on the vertical section of each sample, and the average was calculated.

### 4.3 Property Tests

As shown in Figure 5b, dogbone‐shaped tensile specimens with an original gauge length of 20 mm, gauge width of 10 mm, and thickness of 2 mm were machined from the Cu–Sn alloy with directionally grown grains using electrical discharge machining. The specimen dimensions were in accordance with GB/T 228–2002. The prepared specimens were sanded and cleaned prior to testing. Tensile tests were performed at room temperature using a SUNS‐TM4000 microcomputer‐controlled electronic universal testing machine, with the beam displacement rate set to 1 mm/min. Nb_3_Sn superconducting films of various compositions were deposited onto Cu–Sn alloy substrates via physical vapor deposition (PVD). Compared with the conventional bronze method—which often yields low Nb_3_Sn phase purity and introduces impurities—PVD offers better reproducibility and precise compositional control. In this study, high‐purity Nb targets were used to prepare Nb_3_Sn films with a thickness of 2 µm and high phase purity, enabling more accurate and reliable measurements of superconducting properties. The superconducting properties of the films were characterized by the magnetization intensity and temperature (M–T) and magnetic field intensity (M–H) data obtained from the four‐point probe measurement. The samples were loaded into an isothermal liquid‐helium‐cooled cryostat with a temperature resolution greater than 50 mK [66]. The test adopted the zero‐field cooled method. During the M–H curve test, the magnetic field strength was increased at intervals of 20 Oe. During the M–T curve test, the magnetic field strength was maintained at a constant 1000 Oe. The magnetic field was applied perpendicular to the direction of the Nb_3_Sn films.

### 4.4 First Principles Calculations

**FIGURE 13:**
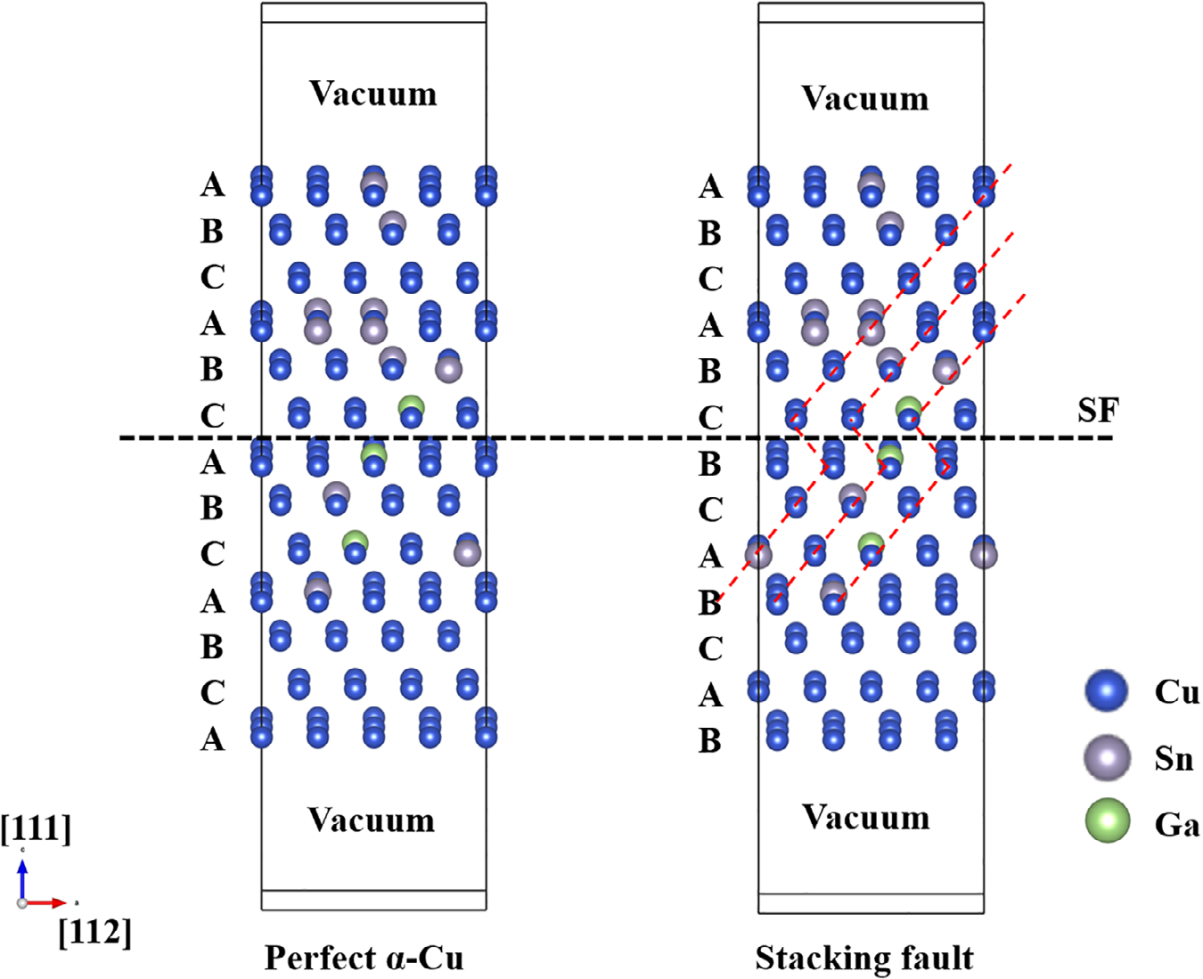
Supercell of the α‐Cu phase with and without SFs.

All first‐principles GSFE calculations were performed using the Vienna Ab initio Simulation Package [67]. The PBE parameterization of the generalized gradient approximation (GGA‐PBE) [68] was used to describe the electronic exchange and correlations. Gamma‐centered *k*‐point meshes [69] were sampled during structural relaxation, that is, in a *k*‐space of 3 × 7 × 1 for the Cu–Sn supercell. The self‐convergence accuracy of the iterative method was set to within 10^−6^ eV, and the force on any atom was less than 0.01 eV/Å. For the pseudopotentials, we chose those recommended for GGA‐PBE. A 2 × 4 × 4 supercell of the Cu supercell with 104 atoms was constructed to model the Cu matrix in this study, as shown in Figure 13, in which the number of Sn atoms was nine, corresponding to mass ratios of 15.0 wt.%, and 1, 2, and 3 Ga atoms were selected in sequence to randomly replace the Cu and Sn sites. Depending on the symmetry operation in the system, duplicate configurations were eliminated by using the *Supercell* program [70]. Ideal solid‐solution configurations were achieved by using the special quasi‐random structure optimization implemented in the Alloy Theoretic Automated Toolkit software [71]. Sharing the (111) plane in a perfect structure with a fault of *a*/6[112] along the <112> direction produces a deformation fault. The GSFE of the SFs containing the Cu supercell as constructed above can be calculated as follows:

(18) $$\gamma_{SFE}=\frac{E_{SF}-E_{Perfect}}{A}$$

where *E_SF_* and *E_Perfect_* correspond to the structures with and without an intrinsic stacking fault, respectively, and A is the area of the fault plane.

Various moduli, Poisson's ratio, formation energy, and other factors of the α‐Cu matrix, Nb_3_Sn substitution model, and Cu–(Ga, Sn) substitution model were calculated using first principles based on density functional theory. These calculations were performed using the Materials Studio software suite. The interaction between ions and electrons was modeled using the projector augmented‐wave method, with the plane wave cutoff set at 400 eV. For the 32‐atom supercell, Brillouin‐zone sampling was performed using a 4 × 4 × 4 k‐point mesh.

## Author Contributions

Dazhuo Song contributed to investigation, data curation, formal analysis, writing of the original draft, and writing – review and editing. Juntao Zou contributed to project administration, supervision, resources, and methodology. Jiayue Zhang contributed to the investigation. Xinhang Liang contributed to writing, review, and editing. Mengyu Shan contributed to data curation. Yifan Liu contributed to validation. Jingqi Feng, Tong Dang, and Lin Shi contributed to data curation. Yuxuan Wang contributed to validation. Yuchen Song contributed to resources. Rong Fei contributed to supervision and resources. Shaodong Sun and Zhe Zhang contributed to the software. Lei Zhu contributed to validation. Lixing Sun contributed to resources.

## Conflicts of Interest

The authors declare no conflicts of interest.

## Data Availability

The data that support the findings of this study are available from the corresponding author upon reasonable request.
